# Biochemical and structural elucidation of the L-carnitine degradation pathway of the human pathogen *Acinetobacter baumannii*

**DOI:** 10.3389/fmicb.2024.1446595

**Published:** 2024-08-14

**Authors:** Fabian Piskol, Peer Lukat, Laurin Kaufhold, Alexander Heger, Wulf Blankenfeldt, Dieter Jahn, Jürgen Moser

**Affiliations:** ^1^Institute of Microbiology, Technische Universität Braunschweig, Braunschweig, Germany; ^2^Department Structure and Function of Proteins, Helmholtz Centre for Infection Research, Braunschweig, Germany; ^3^Institute of Biochemistry, Biotechnology and Bioinformatic, Technische Universität Braunschweig, Braunschweig, Germany; ^4^Braunschweig Centre of Integrated Systems Biology, Technische Universität Braunschweig, Braunschweig, Germany

**Keywords:** *Acinetobacter baumannii*, carnitine, carnitine monooxygenase, trimethylamine, malic semialdehyde dehydrogenase, D-malate dehydrogenase, X-ray crystal structure

## Abstract

*Acinetobacter baumannii* is an opportunistic human pathogen which can use host-derived L-carnitine as sole carbon and energy source. Recently, an L-carnitine transporter (Aci1347) and a specific monooxygense (CntA/CntB) for the intracellular cleavage of L-carnitine have been characterized. Subsequent conversion of the resulting malic semialdehyde into the central metabolite L-malate was hypothesized. Alternatively, L-carnitine degradation via D-malate with subsequent oxidation into pyruvate was proposed. Here we describe the *in vitro* and *in vivo* reconstitution of the entire pathway, starting from the as yet uncharacterized gene products of the carnitine degradation gene operon. Using recombinantly purified enzymes, enantiomer-specific formation of D-malate by the NAD(P)^+^-dependent malic semialdehyde dehydrogenase (MSA-DH) is demonstrated. The solved X-ray crystal structure of tetrameric MSA-DH reveals the key catalytic residues Cys^290^ and Glu^256^, accessible through opposing substrate and cofactor funnels. Specific substrate binding is enabled by Arg^166^, Arg^284^ and Ser^447^ while dual cofactor specificity for NAD^+^ and NADP^+^ is mediated by Asn^184^. The subsequent conversion of the unusual D-malate reaction product by an uncharacterized NAD^+^-dependent malate dehydrogenase (MDH) is shown. Tetrameric MDH is a β-decarboxylating dehydrogenase that synthesizes pyruvate. MDH experiments with alternative substrates showed a high degree of substrate specificity. Finally, the entire *A. baumannni* pathway was heterologously reconstituted, allowing *E. coli* to grow on L-carnitine as a carbon and energy source. Overall, the metabolic conversion of L-carnitine via malic semialdehyde and D-malate into pyruvate, CO_2_ and trimethylamine was demonstrated. Trimethylamine is also an important gut microbiota-dependent metabolite that is associated with an increased risk of cardiovascular disease. The pathway reconstitution experiments allowed us to assess the TMA forming capacity of gut microbes which is related to human cardiovascular health.

## 1 Introduction

**Figure 1:**
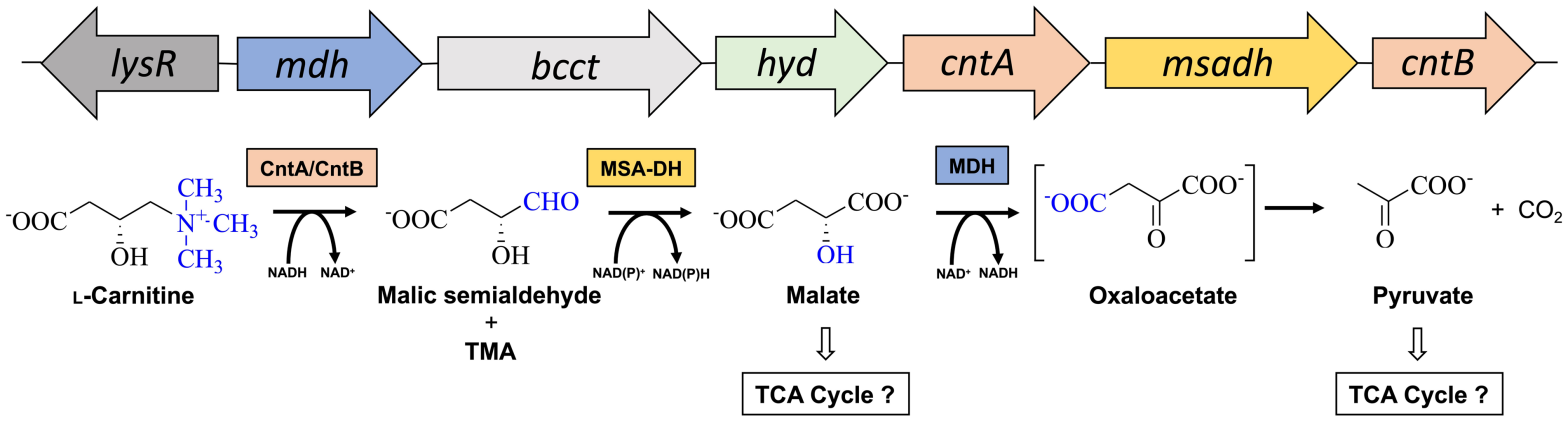
Metabolism of L-carnitine in *A. baumannii*. Structure of the L-carnitine degradation gene operon (*top*) and proposed enzymatic reaction sequence(s) (*bottom*). The cleavage of the C-N bond of L-carnitine is facilitated by carnitine monooxygenase (CntA/CntB), resulting in the formation of trimethylamine (TMA) and malic semialdehyde (Zhu et al., 2014). The latter is converted to malate by malic semialdehyde dehydrogenase (MSA-DH). Pyruvate is formed by the reaction of a potential malate dehydrogenase (MDH). Channeling of reaction products into the central metabolism is indicated. Genes *lysR* (also termed *carR*) and *bcct* encode for a LysR-type transcriptional activator and a transporter of the betaine/choline/carnitine family.

*Acinetobacter baumannii* is an emerging pathogen causing opportunistic infections in healthcare facilities worldwide (Villegas and Hartstein, 2003). The Gram-negative organism is known for the development of high antibiotic resistance and the ability to survive and persist under a wide range of environmental conditions (Dijkshoorn et al., 2007; Ayoub Moubareck and Hammoudi Halat, 2020). *A. baumannii* is a nosocomial pathogen that thrives due to its remarkable ability to withstand desiccation and to persist in the human host (Jawad et al., 1998; Parra-Millan et al., 2018; Zeidler and Muller, 2019). The versatile metabolism of *A. baumannii* facilitates for the utilization of multiple host-derived carbon and nitrogen sources promoting survival in host niches such as the urinary tract, bloodstream or wounds (Dijkshoorn et al., 2007; Soares et al., 2009). Therefore, understanding the employed metabolic pathways is crucial for developing effective strategies to combat infections. One such pathway that might play a vital role in the metabolism of *A. baumannii* is responsible for the degradation for L-carnitine (Zhu et al., 2014; Breisch et al., 2022). This quaternary amine compound is highly available in host tissues (e.g., blood ~50 μM, liver or heart ~1.5 or 5 μmol/g tissue) (Hanai et al., 2020). In the human body, L-carnitine mainly functions as a shuttle for the translocation of long-chain fatty acids into mitochondria for subsequent β-oxidation (Longo et al., 2016). Growth experiments revealed that L-carnitine serves as a sole carbon and energy source of *A. baumannii* (Zhu et al., 2014; Breisch et al., 2022) and a gene cluster for the metabolism of L-carnitine was bioinformatically predicted (Figure 1). Infection studies clearly demonstrated that the corresponding genes are important for the virulence of the opportunistic pathogen (Breisch et al., 2022). The cluster comprises the *carR* gene which encodes for a LysR-type transcriptional activator (*lysR*). L-carnitine dependent binding of the purified CarR protein to the intergenic region between *carR* and the consecutive carnitine catabolic operon was demonstrated (Breisch et al., 2022). Among the components of this operon, the biological function of the encoded transporter Aci01347 was elucidated on the basis of substrate uptake experiments. The transporter belongs to the betaine/choline/carnitine (BCCT) family and mediates the energy-dependent import of L-carnitine but also of choline into the *A. baumannii* cell (Breisch et al., 2019). Furthermore, genes *cntA* and *cntB* of the operon encode for a two-component carnitine monooxygenase which facilitates the initial C-N bond cleavage of L-carnitine which results in the formation of trimethylamine (TMA) and malic semialdehyde (MSA) (Zhu et al., 2014). Our laboratory contributed to the molecular understanding of the catalytic mechanism of the enzyme which comprises the Rieske-type oxygenase CntA and the corresponding reductase CntB (Massmig et al., 2020; Piskol et al., 2022). On the basis of theoretical considerations, it was hypothesized that the operon encodes for a malic semialdehyde dehydrogenase (MSA-DH) which enables for the subsequent conversion of MSA into L-malate which might be channelled into the tricarboxylic acid cycle (Zhu et al., 2014). Alternative L-carnitine degradation via MSA and D-malate with subsequent oxidation into pyruvate by virtue of a D-malate dehydrogenase (MDH) was postulated. Deletion of the *mdh* gene abolished *A. baumannii* growth on L-carnitine which demonstrates the involvement of the postulated MDH in the degradation pathway. Growth of the *mdh* mutant on L-malate was still possible due to the presence of an L-malate dehydrogenase as part of the TCA cycle. Further analysis of the proposed D-malate pathway intermediate was hampered by the possible involvement of a cellular malate racemase and the unavailability of a *msadh* mutant strain. Growth experiments on D-malate for the *mdh* mutant revealed reduced growth (instead of abolished growth) which might be attributed to a theoretical malate racemase activity of *A. baumannii* (Breisch et al., 2022). To date, biochemical evidence for the proposed conversion of L-carnitine via MSA and D-malate into pyruvate and CO_2_ is still pending.

In the present study, we make use of recombinantly purified carnitine monooxygenase, MSA-DH and MDH for the *in vitro* reconstitution of the overall carnitine degradation pathway. Enzyme activities in the presence of variant substrates allow for the elucidation of the (stereo)specificity of the individual reactions. X-ray protein crystallography and/or biochemical experiments are employed for the molecular understanding of the respective enzymes. Finally, the overall pathway is reconstituted under *in vitro* and *in vivo* conditions.

## 2 Materials and methods

### 2.1 Plasmid construction

Constructs pGEX-6P-1-*cntA* and pACYCDuet-1-*cntB* (for the individual production of CntA and CntB) and also plasmid pGEX-6P-1-*cntAB* (for the co-production of CntA and CntB) were described recently (Massmig et al., 2020). Codon-optimized genes for *A. baumannii* ATCC 19606 MSA-DH and MDH were synthesized by GeneArt (Thermo Fisher). Both Genes were cloned via NcoI and NotI restriction sites into plasmid pETM20 and/or pETM11 (Dummler et al., 2005) yielding constructs pETM20-*msadh* or pETM11-*msadh* and pETM11-*mdh.*

For pathway reconstitution experiments, the codon-optimized BCCT gene (Aci1347) was cloned into plasmid pGEX-6P-1-*cntAB* using the In-Fusion cloning system (Takara) yielding construct pGEX-6P-1-*cntAB-bcct*. Plasmid pETM11-cntAB-bcct was obtained by subcloning. cntAB-bcct. The gene for MSA-DH was cloned into MCS1 of pACYCDuet-1 via NotI and NcoI restriction sites and the gene for MDH was cloned into MCS2 via NdeI and XhoI restriction sites yielding pACYCDuet-1-*msadh*-*mdh.*

### 2.2 Production and purification of carnitine monooxygenase

The individual subunits CntA and CntB of carnitine monooxygenase were produced and purified as described elsewhere (Massmig et al., 2020).

### 2.3 Overproduction and purification of MSA-DH and MDH

MSA-DH fused to an N-terminal His-thioredoxin tag or MDH fused to an N-terminal His-tag was overproduced in *E. coli* Tuner (DE3) cells. An overnight culture was used to inoculate 4 × 500 mL of LB medium containing 100 μg ml^−1^ ampicillin (pETM20-*msadh*) or 50 μg ml^−1^ kanamycin (pETM11-*msadh* pETM11-*mdh*), respectively. Cells were cultivated at 37°C and 200 rpm in baffled flasks until an optical density at 578 nm of 0.5 was reached. Subsequently, the production of target proteins was initiated by addition of 50 μM isopropyl-β-d-thiogalactopyranoside (IPTG). After 16 h of cultivation at 17°C and 180 rpm, the cells were harvested by centrifugation at 4,000 × g for 20 min at 4°C. Cells of 2 L culture were resuspended in buffer 1 (100 mM HEPES-NaOH pH 7.5, 10 mM MgCl_2_,150 mM NaCl) or in buffer 1 containing 50 mM NaCl, supplemented with 2.5 units/mL Turbo Nuclease (Jena Bioscience) and disrupted by a single passage through a french press at 14,500 psi. Lysates were clarified by centrifugation at 27,500 × g for 65 min at 4°C and subsequent 0.45 μm filtration. Soluble extracts were applied to 2 mL Ni^2+^-loaded Ni-NTA Agarose (MACHEREY-NAGEL). Columns were washed with 8 mL buffer 1, 8 mL buffer 1 containing 50 mM imidazole and 8 mL buffer 1 containing 100 mM imidazole. Proteins MSA-DH or MDH were eluted with 3 × 2 mL buffer 1 containing 150 mM imidazole. Target protein fractions were identified by SDS-PAGE.

### 2.4 Dialysis of MDH

The apo form of MDH was obtained by EDTA treatment. Purified MDH (~1 mL) was dialyzed for 4 h in 1 L buffer 2 (20 mM MOPS-KOH pH 7.5, 150 mM NaCl) containing 10 mM EDTA followed by three times dialysis against 1 L of buffer 2.

### 2.5 Determination of protein concentration

The concentration of purified proteins was determined using Bradford reagent (Sigma-Aldrich) according to the manufacturer’s instructions with bovine serum albumin as a standard.

### 2.6 Carnitine monooxygenase activity assays

The standard L-carnitine depletion assay was performed as detailed under (Piskol et al., 2022) in the presence 2 mM L-carnitine and 2 mM NADH. An alternative carnitine monooxygenase assay was developed which monitors the time-dependent formation of the reaction product MSA. The assay is based on the determination of aldehydes using 3-methyl-2-benzothiazolinone hydrochloride (MBTH) (Cummins and Hauser, 1964; Zurek and Karst, 1997). A typical 400-μl standard aldehyde formation assay was performed in 20 mM HEPES-NaOH pH 7.5, 10 mM MgCl_2_, 150 mM NaCl containing 5 μM purified CntA, 15 μM purified CntB, and 2 mM L-carnitine at 27°C. Reactions were initiated by the addition of 2 mM NADH. At defined time points (0, 30, 60, 90 and 120 s), 40-μl samples were heat inactivated by the addition of 360 μL pre-heated water (95°C). After 5 min at 95°C followed by centrifugation for 10 min at 12,000 × g, 20 μL of the resulting supernatant was diluted with 80 μL deionized water for subsequent aldehyde determination. A 0.1% (w/v) MBTH solution and an Fe solution consisting of 1% (w/v) iron(III) perchlorate hydrate and 1.6% (w/v) sulfamic acid were each prepared in deionized water. Aldehyde containing samples of 100 μL were mixed with 100 μL MBTH solution on a microplate and incubated at RT for 25 min. Subsequently, 40 μL Fe solution were added and incubated for 30 min before the absorption at 630 nm was determined. Samples with a defined concentration of formaldehyde (2.5 to 50 μM) were processed accordingly. All kinetic experiments were performed in triplicate.

### 2.7 Enzymatic synthesis of MSA

MSA was generated by quantitative turnover of 20 mM L-carnitine in the presence of 20 mM NADH in a 1 mL carnitine monooxygenase reaction as mainly detailed under (Massmig et al., 2020). Resulting aldehyde concentrations were determined as described above. Stoichiometric turnover of the NADH cofactor was verified spectroscopically.

### 2.8 Spectroscopic MSA-DH or MDH activity assays

The activity of MSA-DH or MDH was measured in a continuous spectroscopic assay by determining the initial rate of NADH formation at 340 nm, at a temperature of 27°C using an extinction coefficient of ϵ_340_ = 6.2 mM^−1^ cm^−1^ (Hossain et al., 1984). Standard assays containing 0.2 μM MSA-DH or MDH in 300 μL buffer 1 containing 20 mM NAD(P)^+^ were performed in the presence of 0.5–12 or 20 mM of the respective substrate MSA or D-malate. Experiments (in triplicate) were completed by control reactions in the absence of substrate. Specific activities were determined in the linear range of the assay. Kinetic parameters were calculated according to the Michaelis–Menten equation.

### 2.9 MSA-DH depletion assay

The activity of MSA-DH was also followed by monitoring MSA turnover at 27°C. A typical assay in a volume of 500 μL buffer 1 contained 400 μM MSA, 0.2 μM MSA-DH and 2 mM NAD^+^. At defined time points (0, 30, 60, and 90 s), 40 μL samples were heat inactivated and the aldehyde concentration was determined as detailed above. Experiments in the absence of MSA-DH were processed accordingly. The slope of this control reaction was subtracted to calculate the specific MSA depletion activity in the linear range of the assay. All experiments were performed in triplicate.

### 2.10 Identification of reaction products by HPLC

Samples from MSA-DH assays, from MDH assays or from pathway reconstitution experiments were separated on a Repromer H column (300 × 4,6 mm, 9 μm, TECHLAB GmbH) using a HPLC-system equipped with a degasser (DG-1580-54, Jasco), a gradient unit (LG-1580-04, Jasco), a pump (PU-1580, Jasco), a sampler (AS-1555, Jasco), a column oven (CO-1560, Jasco) and a multi wavelength detector (MD-1515, Jasco). Isocratic separation (~ 50 μL) was performed using 3 mM sulfuric acid as liquid phase at a flow rate of 0.5 mL min^−1^ at room temperature, measuring absorbance at 210 nm. Samples were treated by heat precipitation (99°C, 10 min) followed by centrifugation (12,000 g, 10 min). Authentic malate or pyruvate samples (Sigma-Aldrich) at a concentration of 10 mM were analyzed accordingly. A 50 μL sample from a MSA-DH assay was subjected to preparative separation with subsequent fractionation.

### 2.11 HPLC separation of stereoisomers

The separation of D- and L-malate was performed on a Chirex 3126 (D)-penicillamine column (150 × 4.6 mm, Phenomenex Inc.) using the above chromatography system as detailed mainly under application note TN-1005. Isocratic enantiomer separation was at a flow rate of 1 mL min^−1^ at 27°C using a mobile phase consisting of 85% (v/v) 100 mM ammonium acetate, 1 mM Cu(II)-acetate (pH 4.5, with acetic acid) and 15% (v/v) 2-propanol. The HPLC purified malate fraction from the MSA-DH assay was diluted 1:1 in the mobile phase and subjected to enantiomer separation (25 μL). Authentic L-malate and D-malate samples (5 μL, 10 mM, Sigma Aldrich) were analyzed as a reference.

### 2.12 UV–vis spectroscopy

Purified protein fractions at a concentration of 20–30 μM were subjected to UV–vis spectroscopy using a V-650 UV–Vis spectrophotometer (Jasco).

### 2.13 Analytical size exclusion chromatography

The native molecular mass of MSA-DH and MDH was determined by analytical size exclusion chromatography. A Superdex 200 increase 5/150 GL column (GE Healthcare) was calibrated with protein standards (molecular weight marker kit MWGF200 and thyroglobulin and apoferritin from Sigma) in the presence of 20 mM HEPES-NaOH pH 7.5, 150 mM NaCl at a flow rate of 0.45 mL min^−1^. Samples of MSA-DH and MDH at a concentration of 3 mg mL^−1^ (50 μL) were injected. Elution was monitored at 280 nm.

### 2.14 Mass photometry

The mass photometry experiments were performed on a Refeyn TwoMP mass photometer (Refeyn Ltd). Movies of 60 s (regular FOV) were recorded for 25 nM MDH or 25 nM MSA-DH, respectively in PBS with filtered PBS as a reference using AquireMP (Refeyn Ltd). Evaluation was performed in DiscoverMP (Refeyn Ltd) and molecular masses were determined by matching histogram contrast against a molecular weight standard (Invitrogen/Thermo NativeMark™, LC0725).

### 2.15 Protein crystallography

Crystallization trials were set up at room temperature with a Crystal Gryphon crystallization robot (Art Robbins Instruments) in Intelli 96–3 plates (Art Robbins Instruments) with 200 nL protein solution at a concentration of 40 mg/mL supplemented with 7 mM D-malate and 200 nL reservoir solution. Well-diffracting crystals were obtained after a few days in condition G8 of the Pi-PEG screen (Jena Bioscience), containing 34.3% (w/v) PEG 550 monomethyl ether and 2.9% (w/v) PEG 300. Crystals were after harvesting flash cooled in liquid N_2_.

Data collection was performed at beamline P11 at the Petra III storage ring (Deutsches Elektronen-Synchrotron, Hamburg, Germany) (Burkhardt et al., 2016). Datasets were recorded at a temperature of 100 K. Data processing was carried out using the AutoPROC (Vonrhein et al., 2011) toolbox (Global Phasing) executing XDS (Kabsch, 2010), Pointless (Evans, 2006), and Aimless (Evans and Murshudov, 2013).

The structure of MSA-DH was determined by molecular replacement using a model generated by Alpha-Fold2 (Jumper et al., 2021) as a search-model for Phaser (McCoy et al., 2007) from the Phenix suite (Adams et al., 2010). The structural models were built using Coot (Emsley et al., 2010) and crystallographic refinement was performed with Phenix.refine (Afonine et al., 2012) including the addition of hydrogens in riding positions and TLS-refinement. 5% of random reflections were flagged for the calculation of R_free_. The model of MSA-DH was at 2.6 Å resolution refined to R/R_free_ of 18/21% in space group P2_1_2_1_2_1_. The structural model contains 12 polypeptide chains in the asymmetric unit, corresponding to three tetramers of MSA-DH. Data collection and refinement statistics are summarized in Supplementary Table S1.

NADP^+^ as a ligand was extracted from *E. coli* succinic semialdehyde dehydrogenase [PDB: 3JZ4, (Langendorf et al., 2010)] after structural superimposition with MSA-DH. MSA as a ligand was generated by addition of a hydroxy group to succinic semialdehyde (SSA) using Maestro (Schrödinger Suite 2023–4, Schrödinger, LLC), subsequent to the extraction of SSA from human SSA-DH [PDB: 2W8Q, (Kim et al., 2009)] superimposed with MSA-DH. The crystal structure including both added ligands was then energy minimized using Maestro. Figures of crystal structures were prepared using the PyMOL Molecular Graphics System version 2.4.0 (Schrödinger, LLC). Cavities were calculated using CavitOmiX (v. 1.0, 2022, Innophore GmbH). The corresponding hydrophobicity module of the program VASCo (Steinkellner et al., 2009) was used to analyze the hydrophobicity of the cavities. The cavities were calculated using a modified LIGSITE algorithm (Hendlich et al., 1997).

### 2.16 Pathway reconstitution in *E. coli*

*E. coli* BL21 carrying IPTG inducible plasmids pETM11-*cntAB*-*bcct* and pACYCDuet-1-*msadh*-*mdh* was used for the heterologous *in vivo* reconstitution of the overall carnitine degradation pathway of *A. baumannii*. Cells from a 50 mL overnight culture in LB medium were harvested by centrifugation (4,000 g, 10 min) and resuspended in 20 mL MOPS minimal medium (Neidhardt et al., 1974). The cell suspension was used to inoculate 50 mL of 20 mM L-carnitine supplemented MOPS minimal medium (OD_578_ ~ 0.2) containing 50 μg mL^−1^ ampicillin (pGEX-6P-1-*cntAB*-*bcct*), 17 μg mL^−1^ chloramphenicol (pACYCDuet-1-*msadh*-*mdh*) and 500 μM IPTG. *E. coli* strains BL21(DE3), B and K12 served as a control. Growth experiments were reproduced with three independent cultures.

## 3 Results

### 3.1 Recombinant production and purification of carnitine monooxygenase and MSA-DH

**Figure 2:**
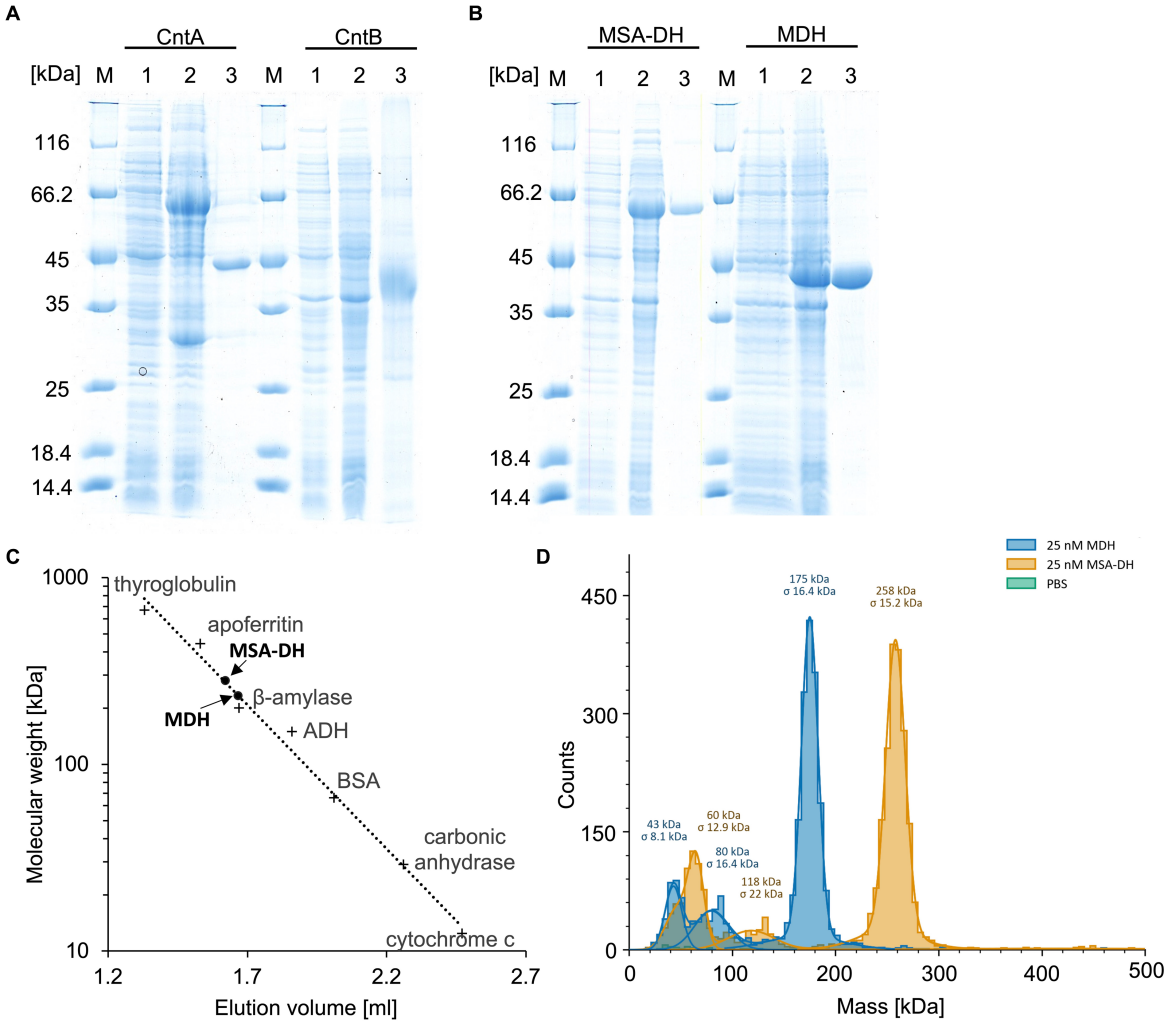
Purification and characterization of the enzymes of the L-carnitine degrading pathway. **(A,B)** SDS-PAGE analysis of the affinity purification of carnitine monooxygenase subunits CntA and CntB, MSA-DH and MDH. *Lanes 1 and 2*, *E. coli* cells before and after IPTG induction; *lanes 3*, elution fractions after cell disruption, ultracentrifugation and affinity chromatography; *lanes M*, molecular mass marker, relative molecular masses are indicated. **(C)** Native molecular mass determination of MSA-DH and MDH by analytical gel filtration. Purified proteins were analyzed on a Superdex 200 increase 5/150 GL column at a flow rate of 0.45 mL min^−1^ monitoring the absorption at 280 nm. The column was calibrated with thyroglobulin (Mr = 660,000), apoferritin (Mr = 440,000), ß-amylase (Mr = 200,000), alcohol dehydrogenase (ADH, Mr. = 150,000), albumin (Mr = 66,000), carbonic anhydrase (Mr = 29,000) and cytochrome c (Mr = 12,400). **(D)** Molecular mass histograms of MSA-DH and MDH obtained from mass photometry measurements using a Refeyn TwoMP instrument. The average molecular mass is given.

The catalytic component CntA and the related reductase CntB of carnitine monooxygenase from *A. baumannii* were individually produced and purified as N-terminal GST-tagged and His-tagged fusion proteins. Proteolytic on-column cleavage of the CntA fusion was performed with PreScission protease (SDS-PAGE Figure 2A, lanes 1–3, *left* and *right*) (Massmig et al., 2020).

Overproduction of MSA-DH in *E. coli* as an N-terminal His-thioredoxin fusion yielded an additional protein band with a relative molecular mass of ∼64,000 Da (calculated molecular mass, 66,592 Da). The recombinant *A. baumannii* protein was affinity purified using Ni^2+^-loaded chelating Sepharose (SDS-PAGE Figure 2B, lanes 1–3 *left*). Approximately 30 mg purified MSA-DH was obtained per liter cell culture.

### 3.2 New carnitine monooxygenase assay

Published carnitine monooxygenase assays rely on the enzymatic quantification of the L-carnitine substrate (Massmig et al., 2020; Piskol et al., 2022) or on the spectroscopic quantification of the co-substrate NADH (Quareshy et al., 2020). These depletion assays either require a coupled detection reaction or, on the other hand, may be affected by a nonproductive NADH consumption (Piskol et al., 2022). Alternatively, sophisticated GC–MS-based approaches were used for the quantification of TMA (Kalnins et al., 2018).

**Figure 3:**
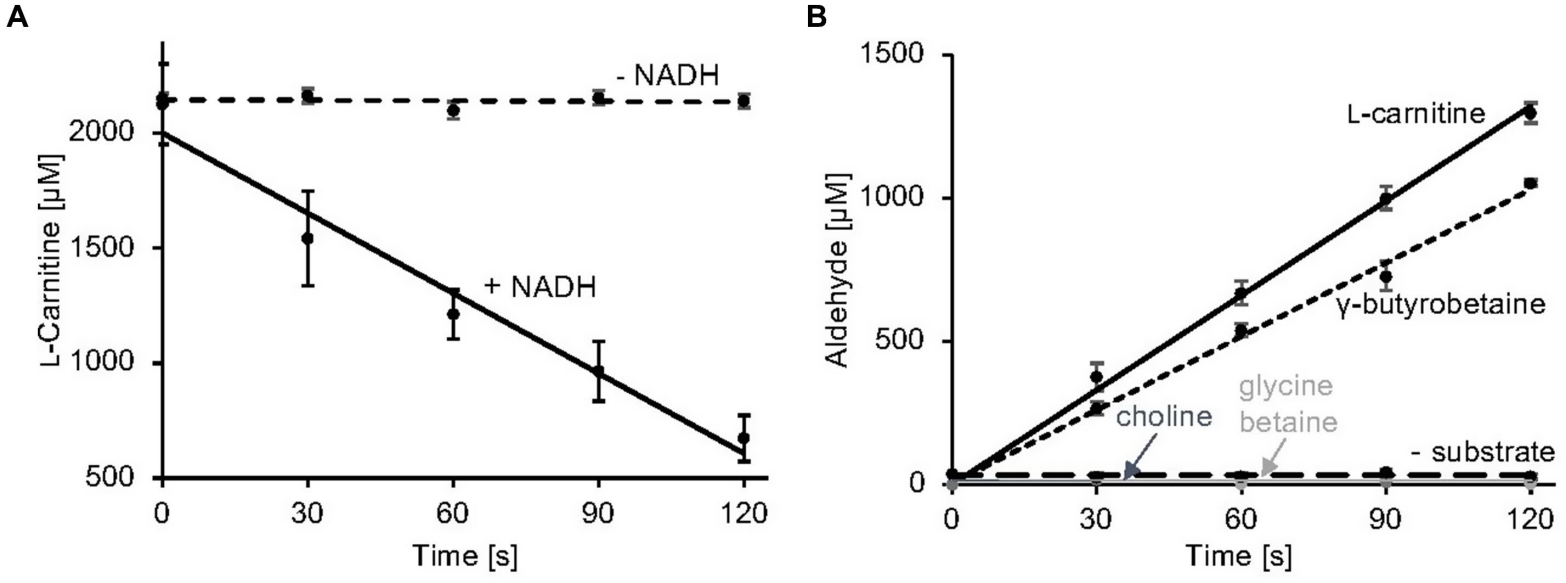
Carnitine monooxygenase activity assays. **(A)** The standard L-carnitine depletion assay containing purified CntA and CntB was initiated by the addition of NADH. L-carnitine concentrations were quantified colorimetrically in a coupled carnitine acetyltransferase reaction as recently described (Piskol et al., 2022) R^2^ values: 0.98 (−NADH), 0.96 (+NADH). **(B)** The newly established carnitine monooxygenase assay monitors MSA production spectrophotometrically using 3-methyl-2-benzothiazolinone (MBTH). Aliphatic aldehyde concentrations were determined in a microplate reader at a wavelength of 630 nm. A control reaction was performed in the absence of substrate. High specific activity was also observed in the presence of the alternative substrate γ-butyrobetaine. No detectable activity was measured in the presence of choline or glycine betaine. R^2^ values: 0.99 (L-carnitine), 0.98 (γ-butyrobetaine), 0.99 (choline), 0.99 (glycine betaine), 0.99 (− substrate). Data shown as mean ± SD from three independent experiments.

Here, a simple and efficient assay was established which is based on the quantification of the second reaction product of carnitine monooxygenase (Figures 3A,B). MSA formation was monitored using 3-methyl-2-benzothiazolinone (MBTH). This reagent enables for the spectrophotometric quantification of aliphatic aldehydes at 630 nm in a microplate reader (Cummins and Hauser, 1964; Zurek and Karst, 1997). Formaldehyde samples of a defined concentration were used to calibrate the system. In Figure 3B, the linear time course of the established carnitine monooxygenase assay is shown which yields a specific activity of 2.8 ± 0.2 μmol min^−1^ mg^−1^. A closely related value of 2.7 ± 0.4 μmol min^−1^ mg^−1^ was calculated on the basis of the well-established L-carnitine depletion assay (Figure 3A) [previously calculated value of 771 nmol min^−1^ mg^−1^ (Massmig et al., 2020)]. The new assay was used for the analysis of the substrate profile of the *A. baumannii* carnitine monooxygenase. Kinetic experiments in the presence of γ-butyrobetaine revealed a high activity of 2.1 ± 0.2 μmol min^−1^ mg^−1^ which is in agreement with a recently published structural investigation (Quareshy et al., 2020). The observed aldehyde formation suggests an analogous reaction mechanism for the conversion of L-carnitine and γ-butyrobetaine. No detectable activity was observed in the presence of choline or glycine betaine (Figure 3B). From this data we conclude that MBTH-based kinetic experiments are a good alternative to the kinetic determinations described so far.

### 3.3 Characterization of MSA-DH

UV–visible absorption spectroscopy of concentrated protein samples did not show the presence of a chromophoric cofactor. Analytical size exclusion chromatography revealed a native molecular mass of 281 kDa (Figure 2C), indicative for a tetrameric quaternary structure of MSA-DH (266 kDa calculated theoretically). However, the methodology used is primarily based on the Stokes radius of the analyzed (fusion) protein. This hydrodynamic value might be strongly influenced by the molecular shape of the MSA-DH fusion protein.

Mass photometry is an alternative experimental technique which allows for the mass measurement of native molecules in solution. Particles are illuminated with a focused laser beam, and their motion is captured by a camera. By analyzing the fluctuations in the scattered light, it’s possible to derive the mass of biomolecules (Soltermann et al., 2020). As indicated in the histogram of Figure 2D, a native mass of 258 kDa for MSA-DH was determined by mass photometry. This result is in good agreement with the analytical size exclusion experiments. Accordingly, *A. baumannii* MSA-DH was found to function as a biological tetramer.

### 3.4 Enzymatic activity of MSA-DH

**Figure 4:**
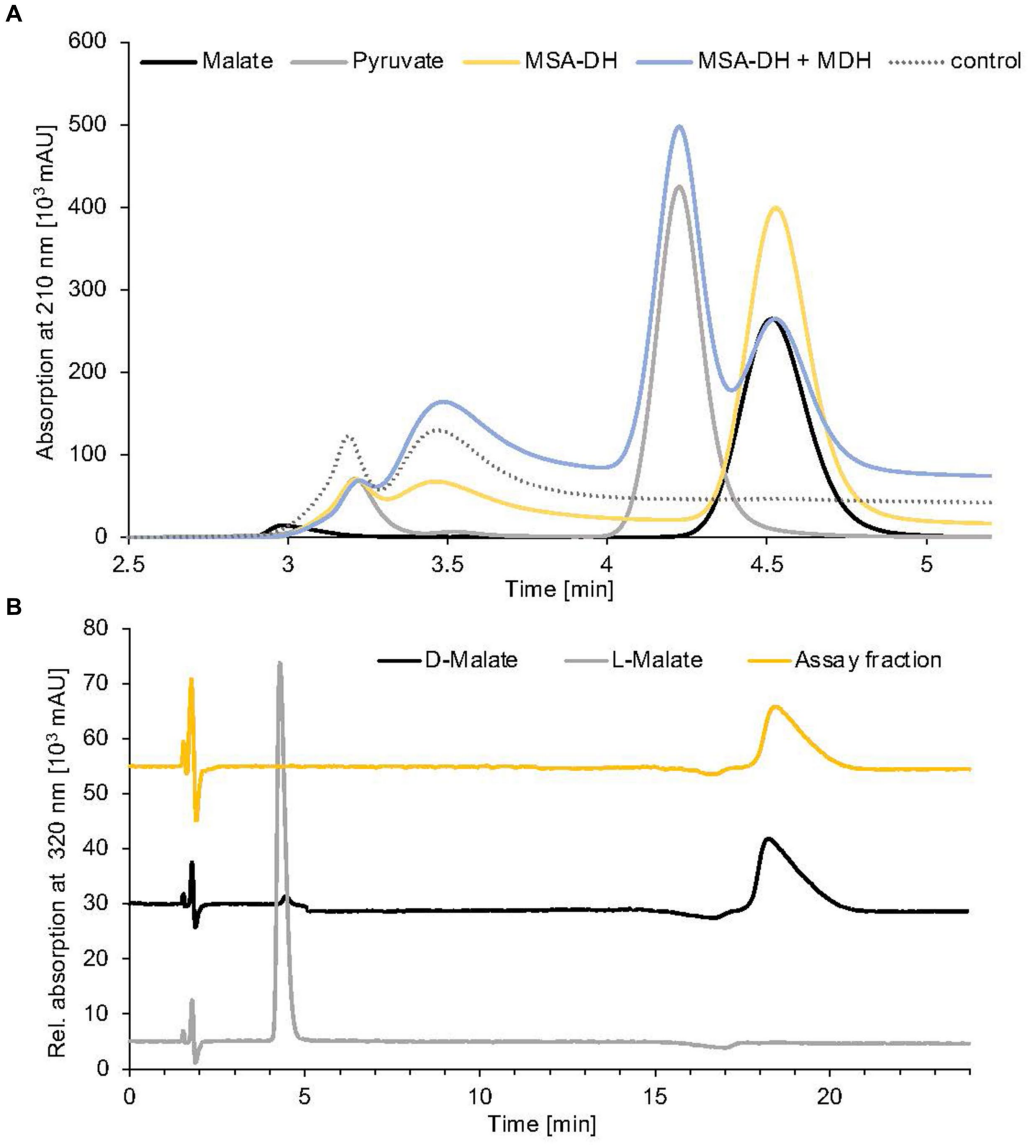
Identification of MSA-DH and MDH reaction products. **(A)** HPLC analysis of pathway reconstitution experiments containing MSA-DH (yellow) or MSA-DH and MDH (blue). The substrate MSA was synthesized *in situ* by the carnitine monooxygenase subunits CntA and CntB. An authentic malate (black) or pyruvate (grey) standard and a control reaction in the absence of MSA-DH and MDH (dotted) were analyzed accordingly. HPLC separation was performed on a Repromer H column using 3 mM sulfuric acid as a mobile phase at a flow rate of 0.5 mL min^−1^ at room temperature. Absorption was monitored at 210 nm. **(B)** Chiral HPLC analysis of the MSA-DH derived reaction product malate. The MSA-DH containing pathway reconstitution experiment from A was further analyzed. The malate-containing fraction was subjected to enantiomer separation on a Chirex 3126 ligand exchange phase (yellow). Authentic D- and L-malate samples were analyzed as standards (black and grey). Isocratic separation was performed at a flow rate of 1 mL min^−1^ at 27°C using 85% (v/v) 100 mM ammonium acetate, 1 mM Cu(II)-acetate (pH 4.5) and 15% (v/v) 2-propanol as a mobile phase.

The aldehyde substrate of the proposed MSA-DH is not commercially available. Therefore, the enzymatic activity of MSA-DH was initially shown in a coupled assay containing carnitine monooxygenase (5 μM CntA and 15 μM CntB) and 5 μM MSA-DH in the presence of the respective cofactors NADH (10 mM) and NAD^+^ (10 mM). By addition of 10 mM L-carnitine, formation of substantial amounts of malate was demonstrated in the HPLC-based approach depicted in Figure 4A (yellow line). Obviously, the reaction of carnitine monooxygenase (or of MSA-DH) is not affected by the presence of high NAD^+^ (or NADH) concentrations. This is a good prerequisite for the intended *in vitro* reconstitution of the overall pathway.

For the subsequent characterization of MSA-DH, substantial amounts of the enzyme substrate were produced enzymatically. The CntA/CntB reaction was carried out on a large scale in the presence of high concentrations of L-carnitine (20 mM) and NADH (20 mM). After an incubation time of 60 min, quantitative substrate and NADH conversion with equimolar MSA production was verified by absorption spectroscopy in combination with the MBTH-based aldehyde quantification method. Subsequent precipitation of CntA/CntB revealed an MSA-containing fraction which was used to provide the enzyme substrate for further biochemical experiments.

**Figure 5:**
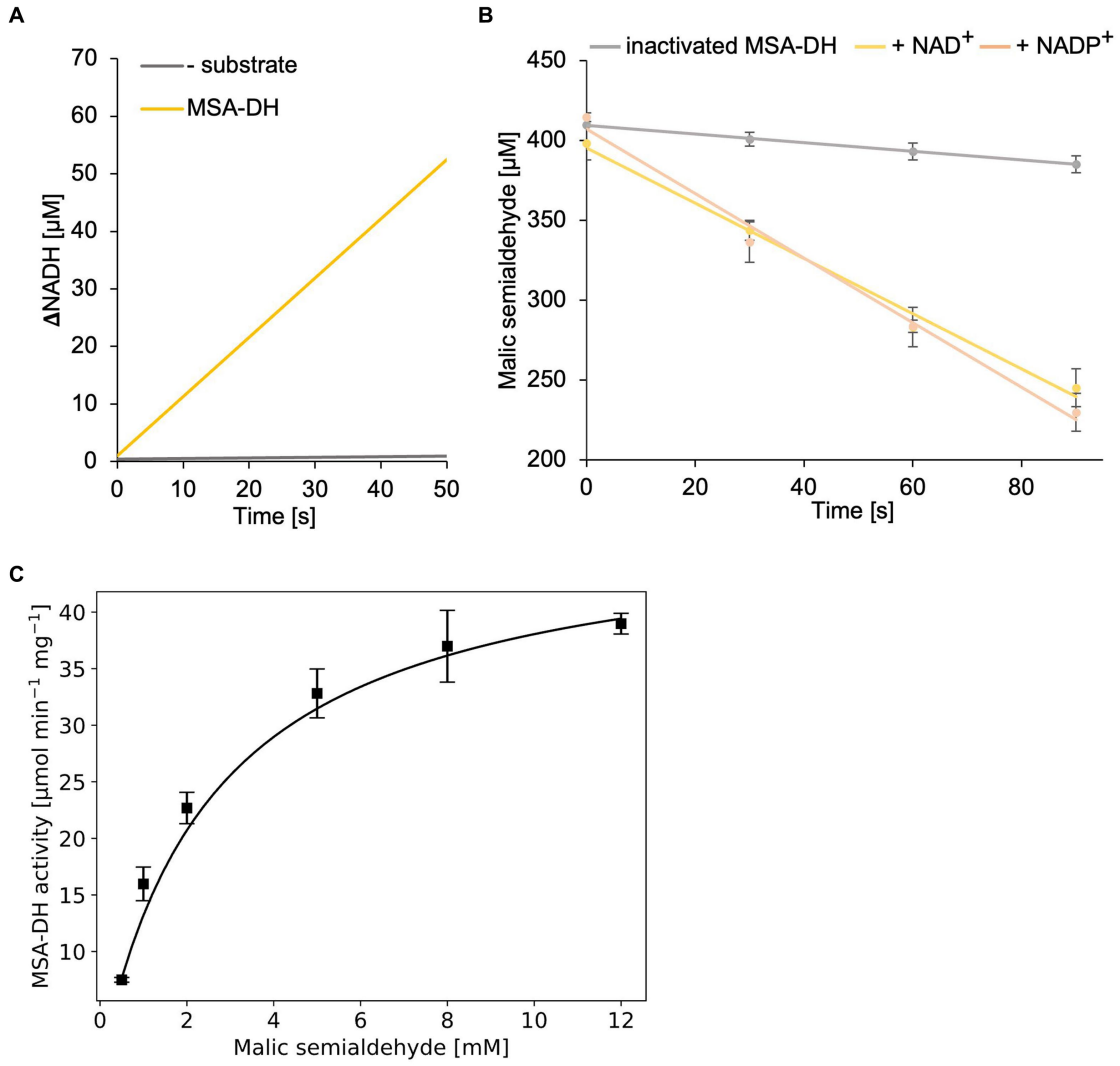
*In vitro* enzyme kinetics and cofactor specificity of MSA-DH. **(A)** Continuous measurement of *in vitro* MSA-DH activity (0.2 μM) in the presence of 400 μM MSA and 20 mM NAD^+^. A linear increase of the NADH concentration (*yellow*) was determined by measuring the absorption at 340 nm. **(B)** MSA-DH dependent depletion of the MSA substrate in the presence of 2 mM NAD^+^ (*yellow*) or NADP^+^ (*light orange*). MSA concentrations were determined spectrophotometrically using the 3-methyl-2-benzothiazolinone (MBTH) method. A control reaction was performed with inactivated MSA-DH (*grey*). R^2^ values: 0.99 (inactivated MSA-DH), 0.99 NAD^+^, 0.99 NADP^+^. **(C)** Michaelis–Menten kinetics of MSA-DH. Initial rates were measured as in A with MSA concentrations ranging from 0.5–12 mM in the presence of 0.2 μM enzyme. R^2^ value: 0.98. **(B,C)** Each point represents the mean of three independent experiments.

In a first MSA-DH *in vitro* assay, substrate-dependent formation of NADH was monitored spectroscopically (Figure 5A). These experiments indicated a pH optimum of pH 7.5–8 in the presence of 400 μM MSA and 2 mM NAD^+^. A specific MSA-DH activity of 7.5 ± 0.2 μmol min^−1^ mg^−1^ was determined. An alternative MSA-DH *in vitro* activity assay was developed which monitors the depletion of the aldehyde substrate using an MBTH-based approach (Figure 5B). A comparable specific MSA-DH activity of 9.7 ± 1.1 μmol min^−1^ mg^−1^ was measured.

MSA-DH activity experiments in the presence of the cofactor NAD^+^ or NADP^+^ (2 mM) revealed closely related enzyme activities (Figure 5B). Thus it was concluded that MSA-DH can use NAD^+^ or NADP^+^ as a cofactor for the oxidation of the MSA substrate under *in vivo* conditions. Experiments with varying substrate concentrations revealed Michaelis–Menten type kinetics with a V_max_ of 48.1 ± 1.4 μmol min^−1^ mg^−1^ and a K_m_ of 2.6 ± 0.14 mM MSA (Figure 5C).

### 3.5 Specific formation of D-malate by MSA-DH

Conversion of MSA into malate in MSA-DH activity experiments was confirmed by standard HPLC experiments (Figure 4A, yellow line). However, the understanding of the stereochemistry of the MSA-DH reaction is crucial for the elucidation of the overall carnitine degradation pathway: L-malate might be directly channeled into the tricarboxylic acid cycle whereas D-malate would require a specific oxidation step that potentially yields the central metabolite oxaloacetate or pyruvate.

Reaction products from a standard MSA-DH assay were fractionated by preparative HPLC separation (compare Figure 4A) and the malate containing fraction was subjected to enantiomeric separation on a Chirex ligand exchange phase 3126. Authentic L-malate or D-malate samples reproducibly eluted at 4.4 min or 18.3 min (Figure 4B, grey and black line). MSA-DH dependent formation of D-malate was indicated at a retention time of 18.4 min (yellow line). Based on these findings, it was concluded that *A. baumannii* MSA-DH catalyzes the stereospecific formation of D-malate which might be further metabolized as part of the degradation pathway.

### 3.6 The three-dimensional crystal structure of MSA-DH

**Figure 6:**
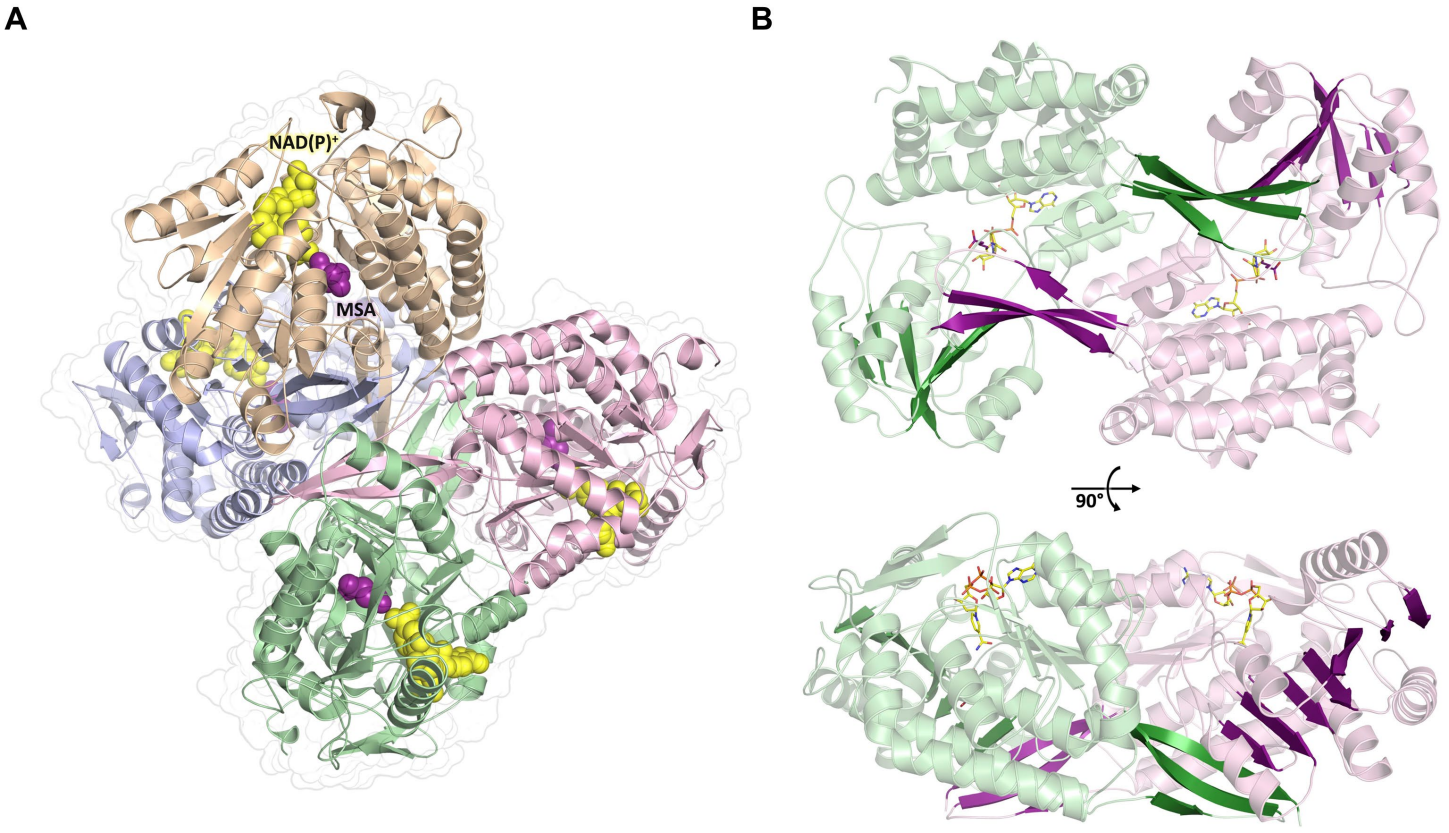
Overall crystal structure of MSA-DH. **(A)** Structure of the native tetramer. *In silico* modeled NAD(P)^+^ and MSA are shown as *yellow* and *purple spheres*. **(B)** Obligate dimer with modeled ligands shown as sticks. The 10-stranded β-sheets formed by domain swapping are highlighted.

To further investigate the molecular mechanism and cofactor utilization of MSA-DH, the X-ray protein structure was determined at 2.6 Å (data collection and refinement statistics in Supplementary Table S1). The asymmetric unit contains an overall of three individual MSA-DH homotetramers which is consistent with our gel filtration and mass photometry experiments. The biological unit is composed of two obligate dimers (e.g., AB and CD colored brown/blue and green/pink) which are arranged to generate a tetramer with almost perfect *D*_2_ symmetry (Figure 6). Corresponding monomers show a root-mean-square deviation (r.m.s.d of all Cα’s) of 0.116 to 0.212 Å.

**Figure 7:**
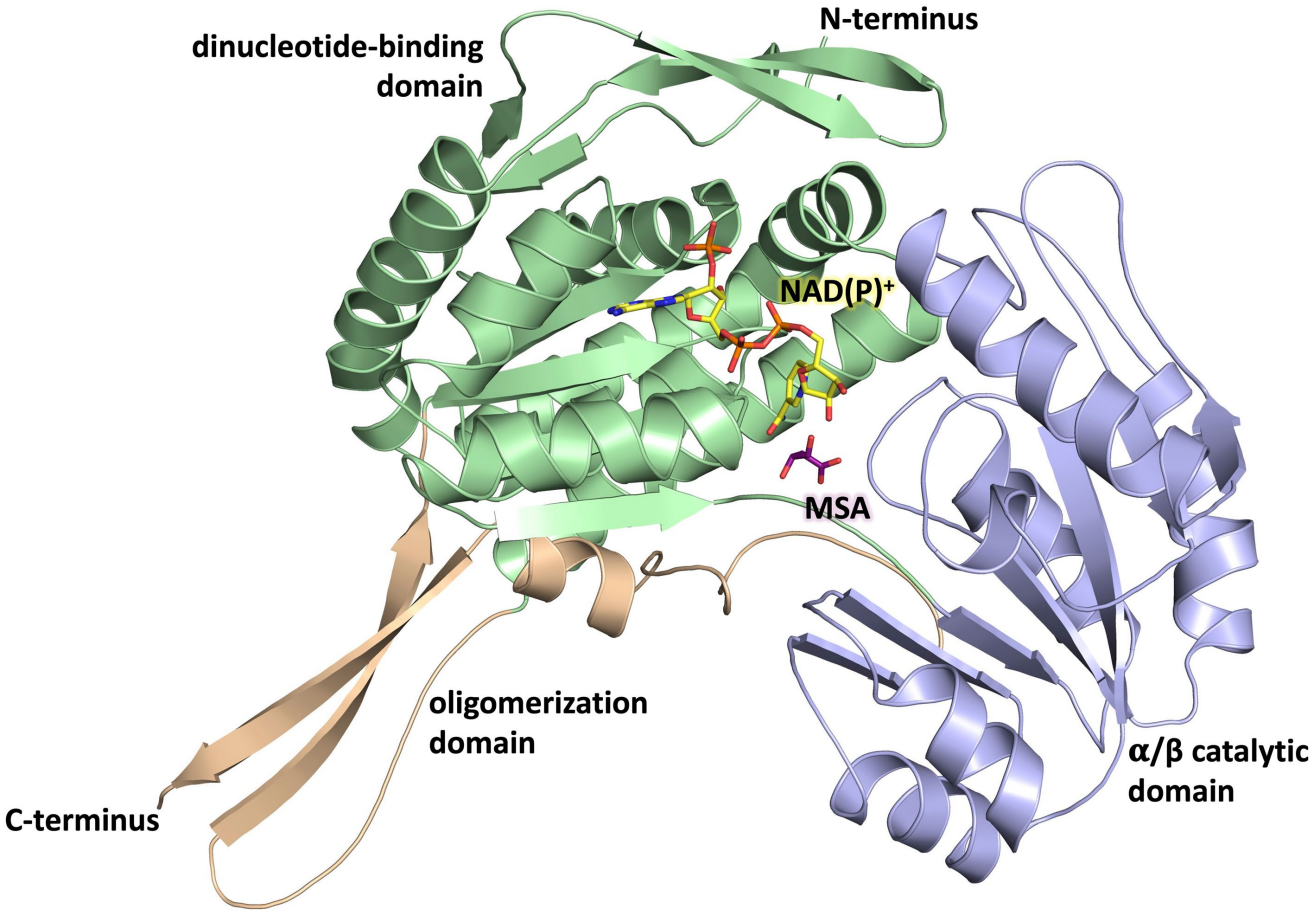
Domain architecture of the MSA-DH monomer. The Rossmann dinucleotide-binding domain is shown in *green* (residues 1–126, 150–261), the α/β catalytic domain is shown in *blue* (residues 262–441) and the oligomerisation domain is shown in *orange* (residues 127–149, 441–483). The *in silico* modeled NAD(P)^+^ and MSA are shown in *yellow* and *purple*.

The overall fold of MSA-DH is similar to other proteins of the aldehyde dehydrogenase family (Shortall et al., 2021). The L-shaped monomer (Figure 7) consists of the N-terminal Rossmann dinucleotide-binding domain (green, residues 1–126 and 150–261), the α/β catalytic domain (blue, residues 262–441), and the oligomerization domain (brown, residues 127–149 and 441–483). The latter part of the molecule is responsible for the domain-swapping dimerization of AB or CD. Three β-strands of the oligomerization domain and seven β-strands from the catalytic domain form a common 10-stranded β-sheet, respectively (Figure 6B, highlighted green and purple).

**Figure 8:**
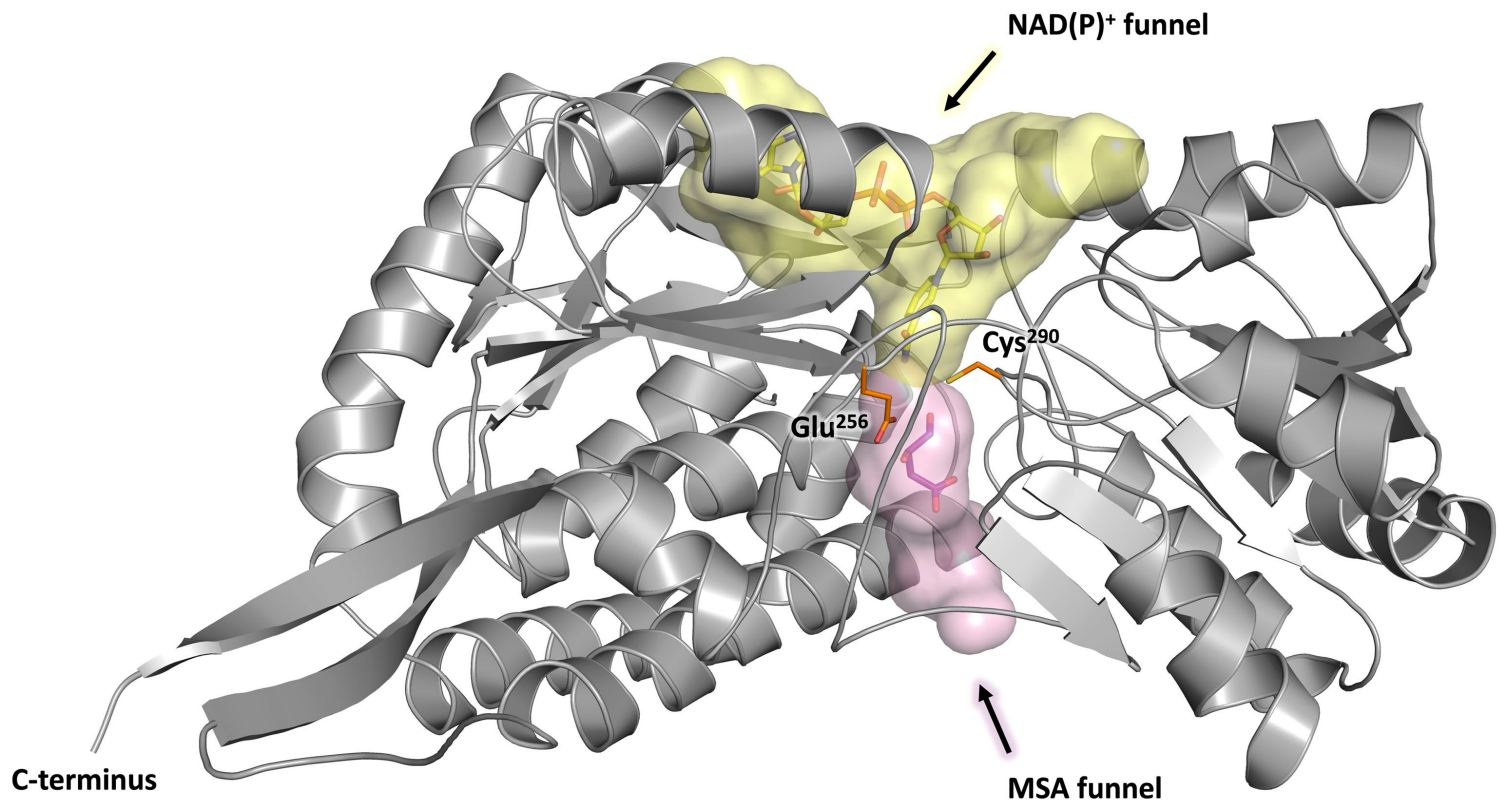
Substrate and co-substrate funnels of MSA-DH. A monomer of MSA-DH (*grey*) with the NAD(P)^+^ funnel (depicted as *yellow spheres*) and the MSA funnel (*purple spheres*) as found by the PyMOL plugin CavitOmiX (v. 1.0, 2022, Innophore GmbH) is shown. NAD(P)^+^ and MSA have been modeled into the corresponding funnels and are shown as *yellow* and *purple sticks*. The key catalytic residues Glu^256^ and Cys^290^ are shown as *orange sticks*.

Every monomer possesses two funel-like invaginations (Figure 8) which were proposed as potential entry sites for the MSA substrate and the cofactor NAD(P)^+^, respectively. Starting from opposite sides, both channels meet in a region where the two highly conserved amino acids Cys^290^ and Glu^256^ are localised. The corresponding cystein and glutamate residues have been described as key catalytic residues of the aldehyde dehydrogenase family (Shortall et al., 2021). Subsequently, the potential cofactor and substrate binding sites of MSA-DH were explored on the basis of structurally related aldehyde dehydrogenase protein structures.

### 3.7 Cofactor utilization of MSA-DH

**Figure 9:**
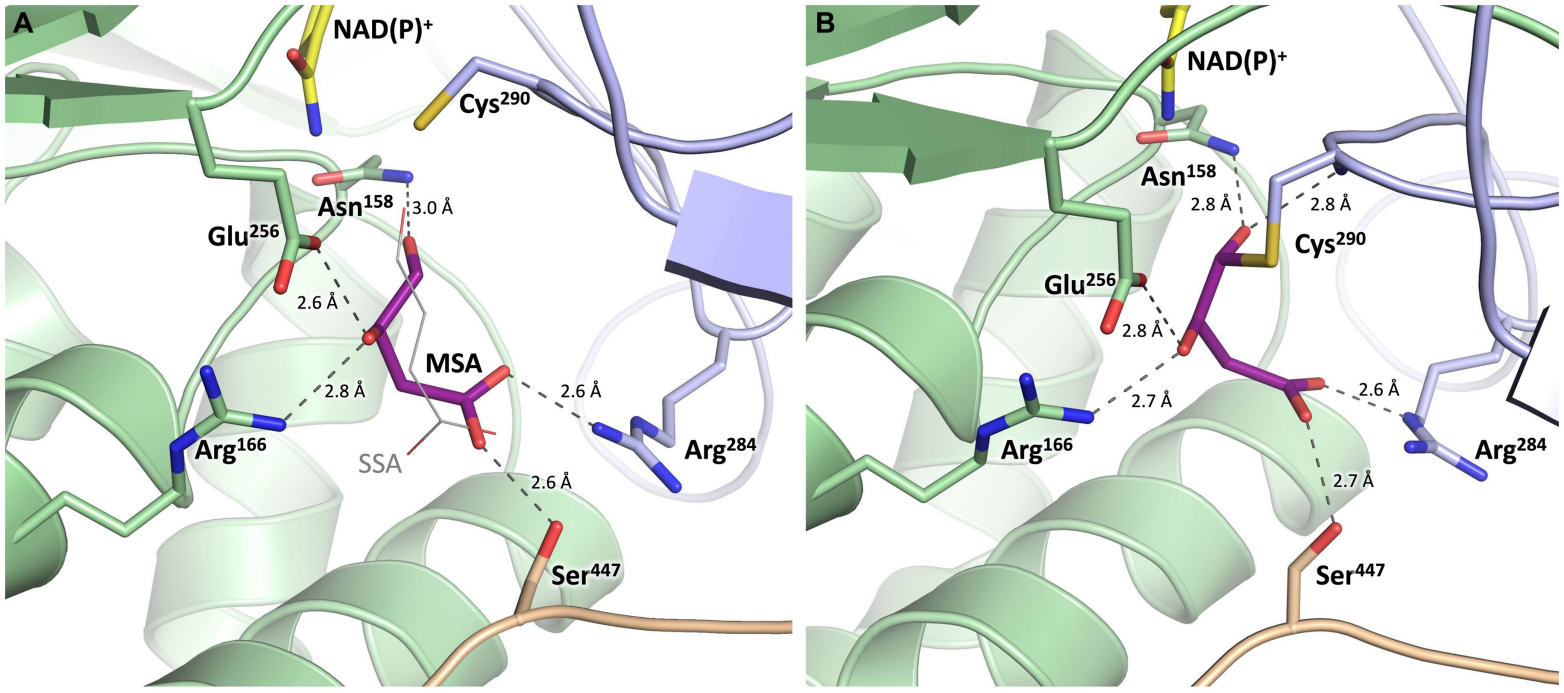
Substrate binding site of MSA-DH with *in silico* modeled ligands. Hydrogen bonds are shown as dashed lines. **(A)** Succinic semialdehyde (SSA, *thin, grey*) was superimposed using human succinic semialdehyde dehydrogenase (2W8Q). MSA (*purple*) was modeled by addition of the 3-hydroxyl group and energy minimization. **(B)** Model of a reaction intermediate (*purple*, modeled from A) covalently bound to Cys^290^.

Coordinates of *E. coli* succinic semialdehyde dehydrogenase in complex with NADP^+^ (PDB ID: 3JZ4) (Langendorf et al., 2010) were structurally superimposed to the native MSA-DH structure (Supplementary Figure S1). As indicated in Figures 7–9, the NADP^+^ molecule was located in a well-defined cofactor binding pocked which did not produce steric clashes. Residues Trp^157^, Lys^181^, Ser^235^ and Glu^387^ of the proposed nucleotide binding pocket (Supplementary Figures S1, S2) of MSA-DH revealed a direct counterpart in the *E. coli* co-crystal structure (residues Trp^155^, Lys^179^, Ser^233^ and Glu^385^). Accordingly, the solved protein structure with the superimposed NADP^+^ molecule was used to assess the cofactor specificity of *A. baumannii* MSA-DH. Based on related structural investigations, one key residue of aldehyde dehydrogenases was identified that is of importance for the recognition (or discrimination) of NAD^+^ and/or NADP^+^ (Yuan et al., 2013). The respective amino acid of MSA-DH was identified as Asn^184^. This asparagine residue does not allow discrimination of the extra phosphate group of NADP^+^ (as observed in the presence of a glutamate). Furthermore, Asn^184^ cannot mediate a favorable interaction with the NADP^+^ phosphate group (as observed in the presence of a serine or threonine). Accordingly, a relaxed NAD(P)^+^ cofactor specificity was expected on the basis of the solved MSA-DH structure. Our structural work thus explains the almost identical MSA-DH activities in the presence of either NAD^+^ or NADP^+^.

### 3.8 Proposed substrate recognition and enzyme mechanism of MSA-DH

The potential substrate binding site of MSA-DH was explored by superimposing the three-dimensional structure of human succinic semialdehyde dehydrogenase in complex with its substrate (PDB ID: 2W8Q) (Kim et al., 2009) (Supplementary Figure S1). Succinic semialdehyde (4-oxobutanoate) acts as a suitable substrate ´analog` as this molecule only lacks the 3-hydroxy group of MSA (3-hydroxy-4-oxobutanoate) which could be added *in silico*.

In Figure 9, the resulting model with MSA (purple) in the proposed active site of MSA-DH is depicted. The *in silico* experiment revealed a well-defined substrate binding pocked that did not cause steric conflicts when the 3-hydroxy group of MSA was added and the model was energy minimized. The obtained active site model was further substantiated as amino acid residues Arg^166^, Arg^284^ and Ser^447^ were located at an appropriate distance for hydrogen bonding interaction with the carboxylate group of the substrate. A very similar network of hydrogen bonds was also observed in the complex structure of the human succinic semialdehyde dehydrogenase (residues Arg^213^, Arg^334^ and Ser^498^, compare Supplementary Figure S1) (Kim et al., 2009).

Subsequently, the enzymatic mechanism of MSA-DH was deduced from the solved X-ray crystal structure with the superimposed MSA and NADP^+^ molecule. As can be seen from Figure 9A, the highly conserved residues Cys^290^ and Glu^256^ are located in a suitable spatial arrangement to the carbonyl of the MSA substrate. Therefore, a typical aldehyde dehydrogenase catalytic mechanism was concluded. Cys^290^ in the active center is poised to nucleophilically attack the MSA substrate which results in the formation of a covalent hemithioacetal. Subsequently, hydride transfer from the covalent intermediate to the NAD(P)^+^ cofactor results in the formation of a thioacyl intermediate (compare the modelled reaction intermediate depicted in Figure 9B). Finally, a water molecule which is deprotonated by Glu^256^ attacks the thioacyl enzyme intermediate. This results in the formation of malate and the regeneration of residue Cys^290^. As also shown in related structural studies, the general base Glu^256^ could further support the hydride transfer step of MSA-DH catalysis (Kim et al., 2009; Vorobieva et al., 2014; Shortall et al., 2021). Supplementary Figure S3 illustrates the spatial interplay of cofactor and substrate in the MSA-DH active site on the basis of the NAD(P)^+^ and the MSA funnel.

For human succinic semialdehyde dehydrogenase, reversible disulfide bond formation of the active site cysteine (Cys^340^) and an adjacent residue (Cys^342^) was described as regulatory redox-switch mechanism. The purified enzyme was completely inactive in the presence of molecular oxygen and the respective crystal structure revealed a substantial rearrangement of the active site loop region. For this enzyme, *in vitro* activity was only observed under reducing conditions. The active site residue Cys^290^ of *A. baumannii* MSA-DH is also neighbored by the fully conserved Cys^292^. Our protein was purified, crystallized and kinetically characterized in the absence of any reducing agents. The solved crystal structure places the active site loop in a conformation which closely resembles the reduced `active state` of human succinic semialdehyde dehydrogenase. Extensive exposure to molecular oxygen, but also the addition of reducing agents did not influence the specific activity of MSA-DH (air exposure for four days, 7.3 ± 0.3 μmol min^−1^ mg^−1^; 10 mM DTT for one h, 7.4 ± 0.3 μmol min^−1^ mg^−1^). Accordingly, our structural and biochemical data do not support a regulatory switch mechanism for the MSA-DH enzyme from *A. baumannii*.

### 3.9 Recombinant production and purification of the postulated MDH

Subsequent investigations were performed to elucidate the involvement of the postulated MDH enzyme in the L-carnitine degradation pathway. *E. coli* cells were used to produce MDH from *A. baumannii* fused to an N-terminal His-tag. SDS-PAGE analysis revealed an induction band with a relative molecular mass of ∼43,000 Da (calculated molecular mass, 44,007 Da). Ni^2+^-loaded chelating Sepharose was used for the affinity purification of the target protein (Figure 2B, lanes 1–3 *right*). A yield of approximately 40 mg purified MDH was obtained per liter cell culture.

### 3.10 Characterization of the proposed MDH

Concentrated protein samples of MDH did not indicate the presence of a chromophoric cofactor as judged bei UV–visible absorption spectroscopy. Analytical size exclusion chromatography revealed a native molecular mass of 234,000 Da, which is in agreement with a tetrameric or a pentameric MDH architecture (Figure 2C). Accordingly, mass photometry was used to verify the quaternary MDH structure. The histogram of Figure 2D indicates a native mass of 175 kDa (calculated native mass, 176,028 Da). These findings clearly show that *A. baumannii* MDH is a functional tetramer.

### 3.11 Enzymatic activity of MDH

The enzymatic activity of the proposed MDH was initially demonstrated in pathway reconstitution experiments. Therefore, a coupled assay containing carnitine monooxygenase, MSA-DH and 5 μM MDH in the presence of the respective cofactors NADH (10 mM) and NAD^+^ (10 mM) was performed (Figure 4A). HPLC analysis revealed L-carnitine dependent formation of malate with the simultaneous appearance of a second dominant peak at a retention time of 4.2 min. By comparison with an authentic sample, the MDH-dependent formation of pyruvate was demonstrated (blue trace). No formation of oxaloacetate was detected in the experiments performed. Thus, it was concluded that *A. baumannii* MDH belongs to the family of β-decarboxylating dehydrogenases which exclusively form pyruvate as a reaction product (Vorobieva et al., 2014).

**Figure 10:**
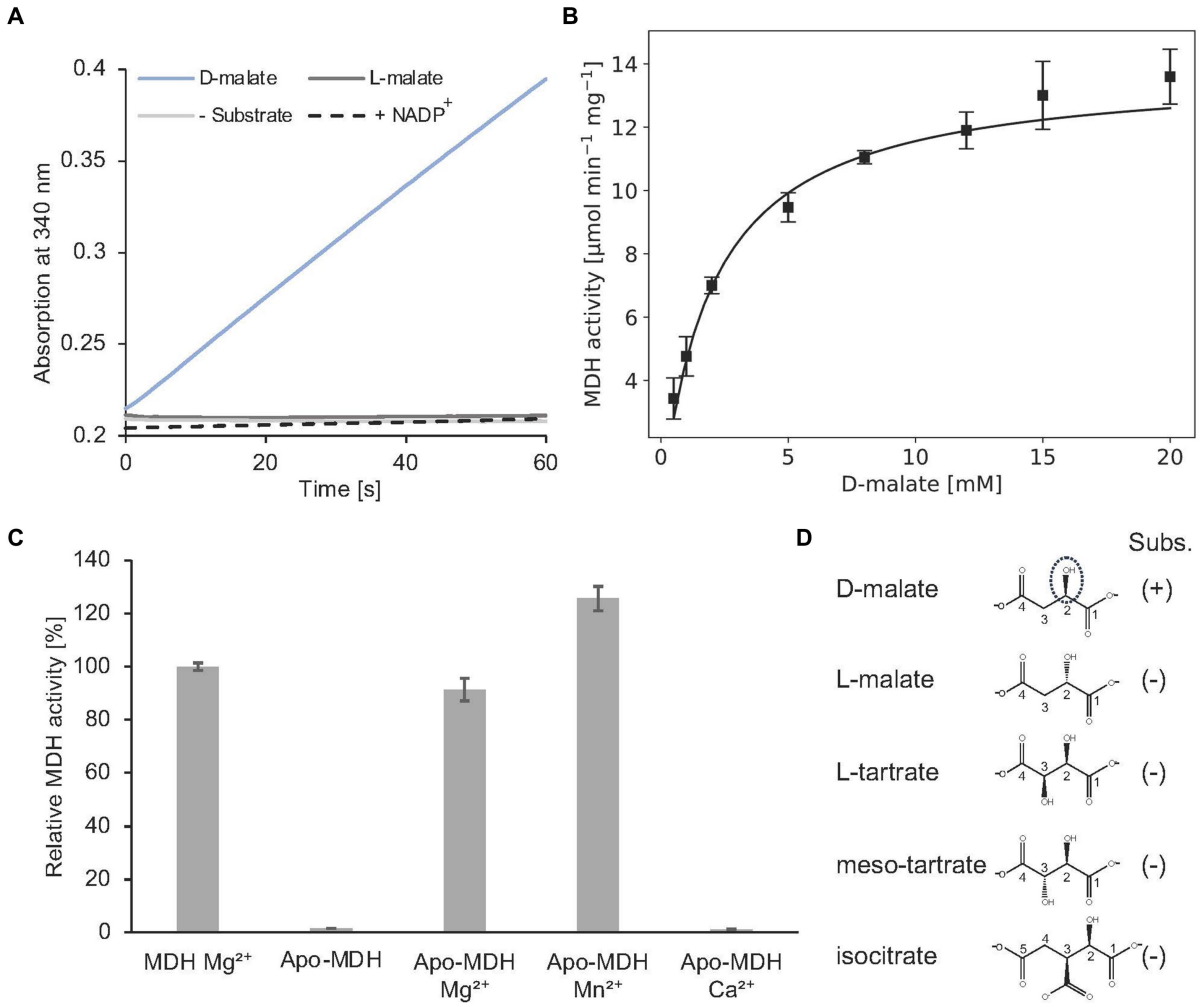
*In vitro* enzyme kinetics, metal cofactor and substrate specificity of MDH. **(A)** Continuous *in vitro* MDH experiments (0.2 μM) in the presence of 400 μM D- or L-malate (*blue* or *dark grey*) and 20 mM NAD^+^. The absorption increase at 340 nm due to the formation of NADH was monitored. A control reaction was carried out without substrate (*grey*). NADP^+^ was tested as an alternative cofactor in the presence of D-malate (*dashed*). **(B)** Michaelis–Menten kinetics of MDH. Initial rates were measured as in A with D-malate concentrations ranging from 0.5–20 mM in the presence of 0.2 μM enzyme. R^2^ value: 0.94. **(C)** Enzymatically active MDH (MDH Mg^2+^) showed a complete loss of enzyme activity after EDTA treatment and dialysis (Apo-MDH), which was efficiently restored by the addition of Mg^2+^ (Apo-MDH Mg^2+^) or Mn^2+^ (Apo-MDH Mn^2+^). Ca^2+^ supplementation did not restore MDH activity (Apo-MDH Ca^2+^). **(D)**, Compounds L-malate, L-tartrate, meso tartrate and isocitrate were tested as alternative substrates of MDH as described under A. **(B,C)**, Each point represents the average of three independent experiments.

Alternatively, MDH activity was followed in a spectroscopic assay which monitors the formation of NADH (Figure 10A). In the presence of 2 mM D-malate and 20 mM NAD^+^, a specific MDH activity of 7.0 ± 0.3 μmol min^−1^ mg^−1^ was determined. Michaelis–Menten type kinetics were determined with a V_max_ of 14.2 ± 0.4 μmol min^−1^ mg^−1^ and a K_m_ of 2.1 ± 0.2 mM for D-malate (Figure 10B).

Complete loss of activity was observed after EDTA treatment and dialysis of MDH. Enzymatic activity of this apo enzyme was fully restored upon Mg^2+^ or Mn^2+^ addition. No MDH activity was observed by Ca^2+^ addition (Figure 10C). From these data a Mg^2+^ or Mn^2+^-dependent catalysis was concluded as also demonstrated for other members of the enzyme superfamily (Hurley et al., 1991; Vorobieva et al., 2014).

### 3.12 MDH catalysis is highly specific

The orthologous D-malate dehydrogenase from *E. coli* (DmlA) was described as a generalist enzyme that contributes to two distinct physiological pathways as indicated by high catalytic activity on alternative substrates (L-tartrate, D-malate, and isocitrate). Substrate promiscuity has been also demonstrated for related enzymes of the superfamily (Miyazaki, 2005b,a).

As indicated in Figure 10A, *A. baumannii* MDH is not able to convert L-malate. Furthermore, NADP^+^ cannot serve as a substitute for the employed NAD^+^ cofactor of MDH. The following compounds with a substitution at the C-3 position were tested as alternative MDH substrates: L-tartrate, *meso*-tartrate and isocitrate (Figure 10D). These molecules did not reveal detectable enzyme activity.

Presence of 2 mM *meso*-tartrate in the standard MDH activity assay (containing 2 mM D-malate) revealed a significant reduction of the enzyme activity (0.93 ± 0.1 μmol min^−1^ mg^−1^). Experiments with varying inhibitor concentrations revealed an inhibition constant K_i_ of 0.32 ± 0.08 mM. Competitive inhibition of MDH in the presence of high *meso*-tartrate concentrations was concluded (Supplementary Figure S4).

In summary, it has been shown that the MDH of *A. baumannii* is a highly specific β-decarboxylating dehydrogenase. Accordingly, catabolism of L-carnitine proceeds via MSA and D-malate and leads to the formation of trimethylamine and pyruvate.

### 3.13 The *cntA* containing gene cluster of *E. coli*

Bacterial L-carnitine metabolism is important for understanding the resilience of potentially pathogenic organisms. However, the overall contribution of the CntA-mediated conversion of L-carnitine has been controversial in the context of microbiome investigations (Zhu et al., 2014; Rath et al., 2018, 2019). For a specific organism, it is not clear whether the presence of the *cntA* gene directly correlates with substantial L-carnitine metabolism and the formation of TMA.

Based on bioinformatics investigations, orthologous *cntA* genes were identified in different genomic contexts (Zhu et al., 2014). The carnitine degradation cluster of *A. baumannii* comprises the LysR-type transcriptional regulator *carR*, *mdh*, the betaine/choline/carnitine transporter gene *bcct*, the gene for a proposed acylcarnitin hydrolase *hyd* which is followed by *cntA*, *msadh* and *cntB*. The related *cntA* containing cluster of *E. coli* only reveals an overall of five orthologous gens: *carR*, *mdh*, *bcct*, *cntA*, and *cntB* showing substantial protein sequence identity of 45, 72, 56, 72 and 52%, respectively (Supplementary Figure S5). The gene cluster clearly differs due to the absence of the *hyd* and the *msadh* gene. However, at a distant site in the genome, *E. coli* also encodes for a reductase protein (GabD) showing 62% identity when compared to *A. baumannii* MSA-DH. Based on these theoretical investigations, it is not clear whether *E. coli* is capable of using the same carnitine degradation pathway as *A. baumannii*. In the current literature, there is no experimental evidence of *E. coli* growth using L-carnitine as the sole carbon and energy source (Jung et al., 1987).

### 3.14 No growth of *E. coli* strains on L-carnitine

High *in vitro* enzyme activity of recombinantly overproduced carnitine monooxygenase from *E. coli* was recently demonstrated (Piskol et al., 2022). However, a manual search of high throughput gene expression data (NCBI GEO database) (Edgar et al., 2002) did not indicate experimental conditions that revealed substantial *cntA* gene transcription under *in vivo* conditions. This was confirmed when the based carnitine monooxygenase assay was performed using a cellular extract of *E. coli* BL21. The laboratory strain after cultivation in complex medium did not indicate detectable carnitine monooxygenase activity.

Subsequently, growth experiments on carnitine containing minimal medium were initiated using different strains of *E. coli*. Strains BL21, B and K12 did not showed measurable growth (Supplementary Figure S6). These initial experiments might indicate that the *cntA* containing operon of *E. coli* serves a different physiological function when compared to the related *A. baumannii* gene cluster. However, these findings show that, e.g., strain *E. coli* BL21 is a suitable platform for the investigation of the *A. baumannii* carnitine degradation pathway.

### 3.15 *In vivo* reconstitution of the carnitine degradation pathway

*E. coli* BL21 carrying two IPTG inducible overexpression plasmids was used for the heterologous *in vivo* reconstitution of the overall carnitine degradation pathway of *A. baumannii*. Overproduction of carnitine monooxygenase subunits CntA and CntB, MSA-DH, MDH and BCCT resulted in a strain that was able to grow slowly on L-carnitine as the sole carbon and energy source (Supplementary Figure S6). After a prolonged lag phase, an OD_578_ of 0.6 was achieved after 24 h of incubation. Carnitine metabolism of this strain was widely recognizable by a distinct TMA odor of the respective culture. Obviously, the alternative metabolic pathway has been successfully implemented in *E. coli*. In order to achieve a more efficient growth of the culture, it may be necessary to have a precisely balanced ratio of the reconstituted protein components. *In vitro* reconstitution of the pathway was only observed upon IPTG induction.

## 4 Discussion

L-carnitine is a quaternary amine compound that serves a variety of physiological functions. Due to its zwitterionic nature, it plays an important role in protecting microbes from osmotic, thermal, cryogenic, and barometric stresses by acting as a compatible solute (Kets et al., 1994; Jebbar et al., 1998; Verheul et al., 1998). Furthermore, L-carnitine has been also described as a final electron acceptor under anaerobic conditions in the presence of additional carbon and nitrogen sources (Seim et al., 1982; Jung et al., 1987; Walt and Kahn, 2002; Meadows and Wargo, 2015).

For *A. baumannii*, a gene cluster for the metabolism of L-carnitine under aerobic conditions was predicted and bacterial growth on L-carnitine as the sole carbon and energy source was demonstrated (Zhu et al., 2014; Breisch et al., 2022). This degradation pathway was also linked to the virulence of the opportunistic pathogen (Breisch et al., 2022). Previous biochemical studies addressed the LysR-type transcriptional activator (*lysR*), the energy-dependent import system BCCT and the two-component carnitine monooxygenase which catalyzes the oxygen-dependent cleavage of L-carnitine into TMA and MSA (Zhu et al., 2014; Breisch et al., 2019; Massmig et al., 2020; Breisch et al., 2022; Piskol et al., 2022). Here we focussed on the remaining pathway enzymes and the funnelling of the reaction intermediates into the central metabolism. The protein crystal structure of MSA-DH in combination with biochemical studies revealed a covalent catalytic mechanism leading to the enantiomer-specific formation of D-malate. The stereochemistry of the reaction product D-malate hampers direct channelling into the tricarboxylic acid cycle. We were able to show that the further required enzyme MDH is a β-decarboxylating dehydrogenase with unusually high substrate specificity. Finally, our *in vivo* and *in vitro* reconstitution experiments clearly demonstrated the conversion of L-carnitine via MSA and D-malate to pyruvate, CO_2_ and TMA.

From the evolutionary perspective, the formation of the unusual D-malate intermediate prevents the direct channeling of malate into the TCA cycle which requires the presence of one further enzyme (MDH). But why has no synthetic pathway evolved that leads directly to the synthesis of L-malate? As can be seen in Figure 1, the stereo center of L-carnitine remains unaltered throughout the individual reaction steps. Neither the enzymatic mechanism of CntA/CntB, nor the reaction sequence of MSA-DH relate to this atom. This shows that a metabolic pathway leading to L-malate would only be possible with the involvement of an additional racemase.

In recent years, the bacterial formation of TMA has attracted much attention due to its medical relevance. TMA has been shown to be a gut microbiota-dependent metabolite that is associated with an increased risk of cardiovascular disease. TMA formed from dietary nutrients such as carnitine or choline is absorbed by the intestinal epithelium and subsequently oxidized to TMAO by hepatic flavin monooxygenases. Several studies have found a positive association between elevated plasma TMAO levels and mortality, particularly deaths due to cardiovascular and renal disease (Kanitsoraphan et al., 2018; Chen et al., 2022). In this context, the capacity of the gut microbiota for TMA production was assessed, e.g., by detecting the presence of the following enzymes: Choline-TMA lyase (*cutC*), betaine reductase (*grdH*) and carnitine monooxygenase (*cntA*) (Falony et al., 2015; Rath et al., 2017, 2019). Based on the results of the present investigation, we propose that *cntA* dependent TMA synthesis should not be judged solely by the presence of the respective gene. We suggest that the genomic context of the *cntA* gene must be also taken into account. In Supplementary Figure S5 the structure of five different types of *cntA* containing operons from representative genomes of human gut microbiota is compared to the carnitine degradation cluster of *A. baumanni* and to the related gene cluster of *E. coli* (Zhu et al., 2014) (A-E). Judging from the results of the present investigation, only the operon structure C and E which is found in *Providencia stuartii* and *Sporosarcina newyorkensis* might facilitate the synthesis of substantial amounts of TMA. Organisms with a *cntA* gene cluster which is devoid of the *msadh* and/or the *mdh* gene as in the operon D of *Achromobacter piechaudii* were expected to mainly use the respective genes for the appropriate adjustment of intracellular L-carnitine concentrations. These organisms might use L-carnitine mainly as a compatible solute to provide protection against various environmental stress conditions. This hypothesis is in line with a previous study showing the absence of *cntA* expression in human fecal samples (Rath et al., 2018) or with another investigation that demonstrated poor correlation between *cntA* abundance and TMAO plasma levels (Wu et al., 2019). Further biochemical studies are required to quantitatively assess the contribution of the encoded TMA-producing enzymes within a given microbiome.

## Data Availability

The datasets presented in this study can be found in online repositories. The names of the repository/repositories and accession number(s) can be found below: Protein structure coordinates are deposited in the Protein Data Bank under the PDB entry code 8S33.
