# Supplementary material for: Biochemical and structural elucidation of the L-carnitine degradation pathway of the human pathogen Acinetobacter baumannii
Source: Front Microbiol. 2024 Aug 14;15:1446595. doi: 10.3389/fmicb.2024.1446595 (PMC11353897; doi:10.3389/fmicb.2024.1446595)
Supplement: Supplementary file 1 [file Table_1.DOCX]

Supplementary Material

# 1 Supplementary Figures and Tables


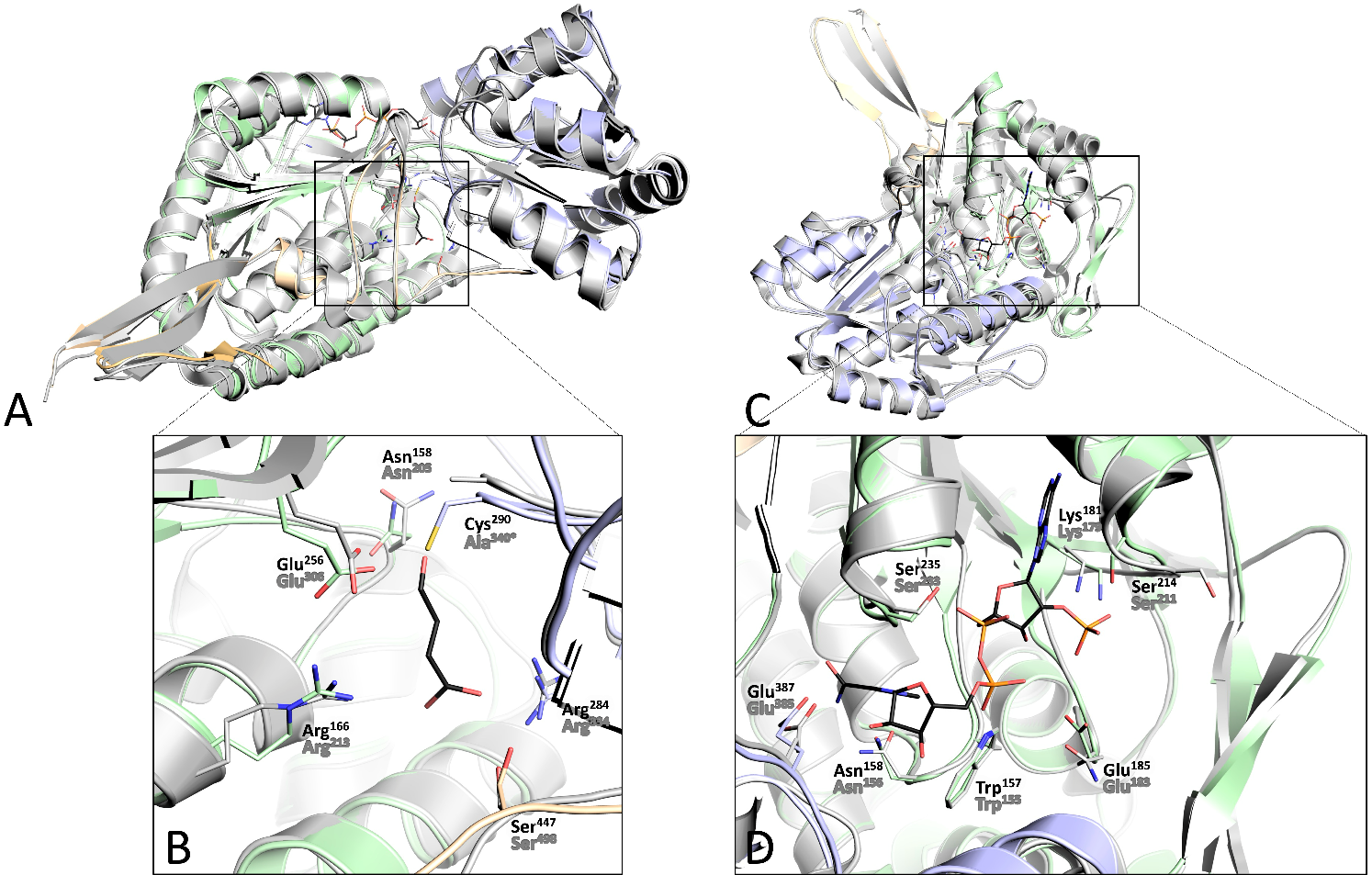


**Figure S1.** **Structural alignment of MSA-DH with** **succinic semialdehyde dehydrogenase from human and *E. coli.* (A),** Superimposition of the MSA-DH crystal structure (domains colored green/blue/orange) with the succinic semialdehyde complex of human succinic semialdehyde dehydrogenase (PDB-ID: 2W8Q, *grey*) and with the NADP^+^ bound form of *E. coli* succinic semialdehyde dehydrogenase (PDB-ID: 3JZ4, *grey*), oriented towards the substrate binding site. **(B)**, Close-up of the substrate binding site of MSA-DH and 2W8Q with succinic semialdehyde shown in *black*. Side chains likely involved in ligand binding in MSA-DH (black labels) and their equivalents in 2W8Q (*grey labels*) are shown as sticks. The asterisk on Ala^340^ of 2W8Q indicates that this residue has been mutated in the PBD entry and is a cysteine in the wild-type. **(C),** Same as panel A, but oriented towards the NADP^+^ binding site. **(D)**, Close-up of the NADP^+^ binding site of MSA-DH and 3JZ4 with NADP^+^ shown in black. Side chains likely involved in NADP^+^ binding in MSA-DH (*black labels*) and their equivalents in 3JZ4 (*grey labels*) are shown as sticks.


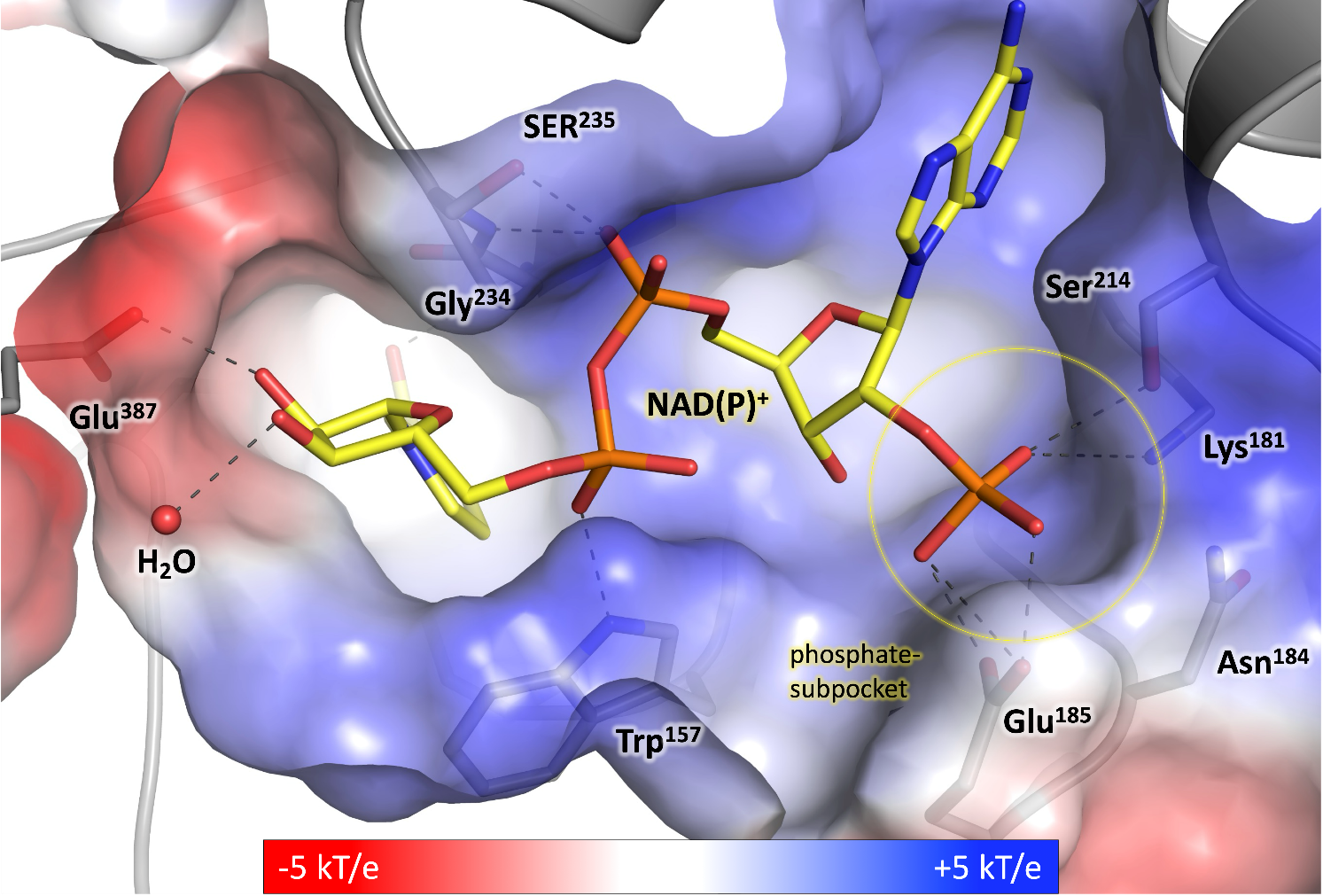


**Figure S2.** **Surface representation of the NAD(P)^+^ binding pocket of MSA-DH.** The cofactor shown was obtained by superimposing the structure of *E. coli* succinic semialdehyde dehydrogenase in complex with NADP^+^ (PDB ID: 3JZ4). Residues Trp^157^, Lys^181^, Ser^235^ and Glu^387^ of the MSA-DH nucleotide binding pocket revealed a direct counterpart in the *E. coli* co-crystal structure. Asn^184^ was identified as a key residue for the relaxed NAD(P)^+^ cofactor specificity of MSA-DH. The proposed phosphate subpocket is highlighted. Surface polarity was calculated using APBS from within PyMOL (1).


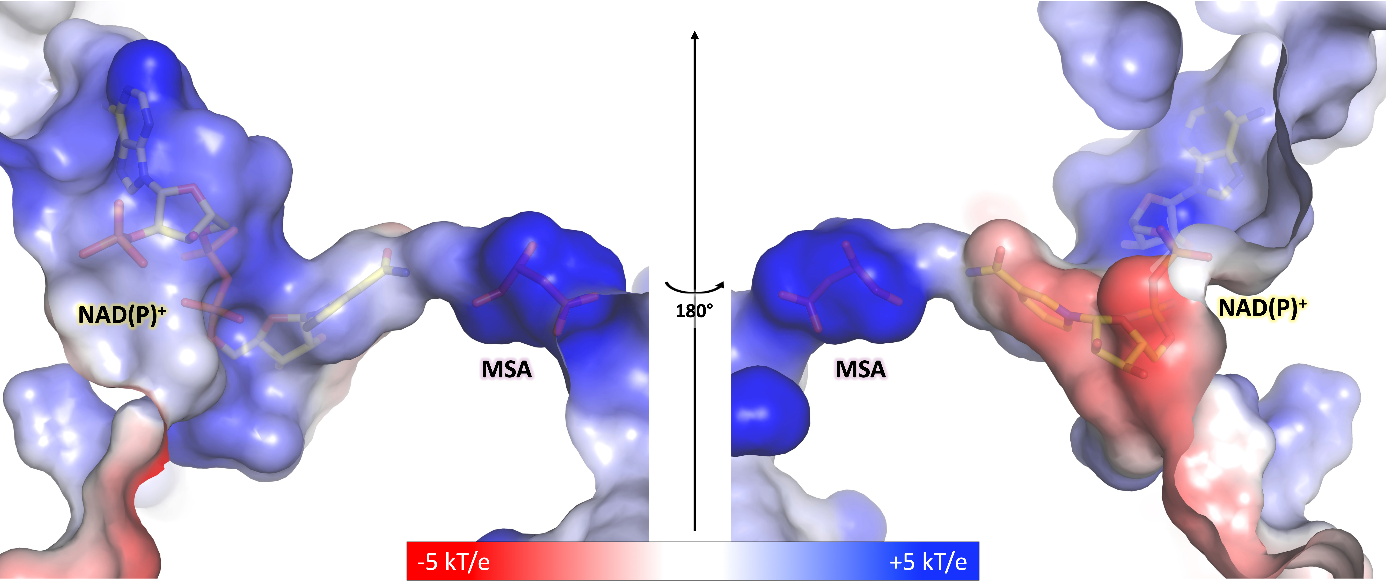


**Figure S3.** **Polar surface representation of the substrate and co-substrate binding site of MSA‑DH.** NAD(P)^+^ and MSA have been modeled into the corresponding binding sites and are shown as *yellow* and *purple sticks*. Surface polarity was calculated using APBS from within PyMOL (1).

**Figure S4.** **Inhibition of MDH by meso-tartrate.** Continuous *in vitro* MDH activity experiments containing 0.2 µM enzyme in the presence of 2 mM NAD^+^, 2 mM D-malate and 2 mM (dark-blue), 200 µM (medium-blue) or 20 µM (light-blue) meso-tartrate, respectively. The absorption increase at 340 nm due to the formation of NADH was monitored. Control experiments in the absence of the substrate D-malate (*grey*) or without meso-tartrate (black) were performed.


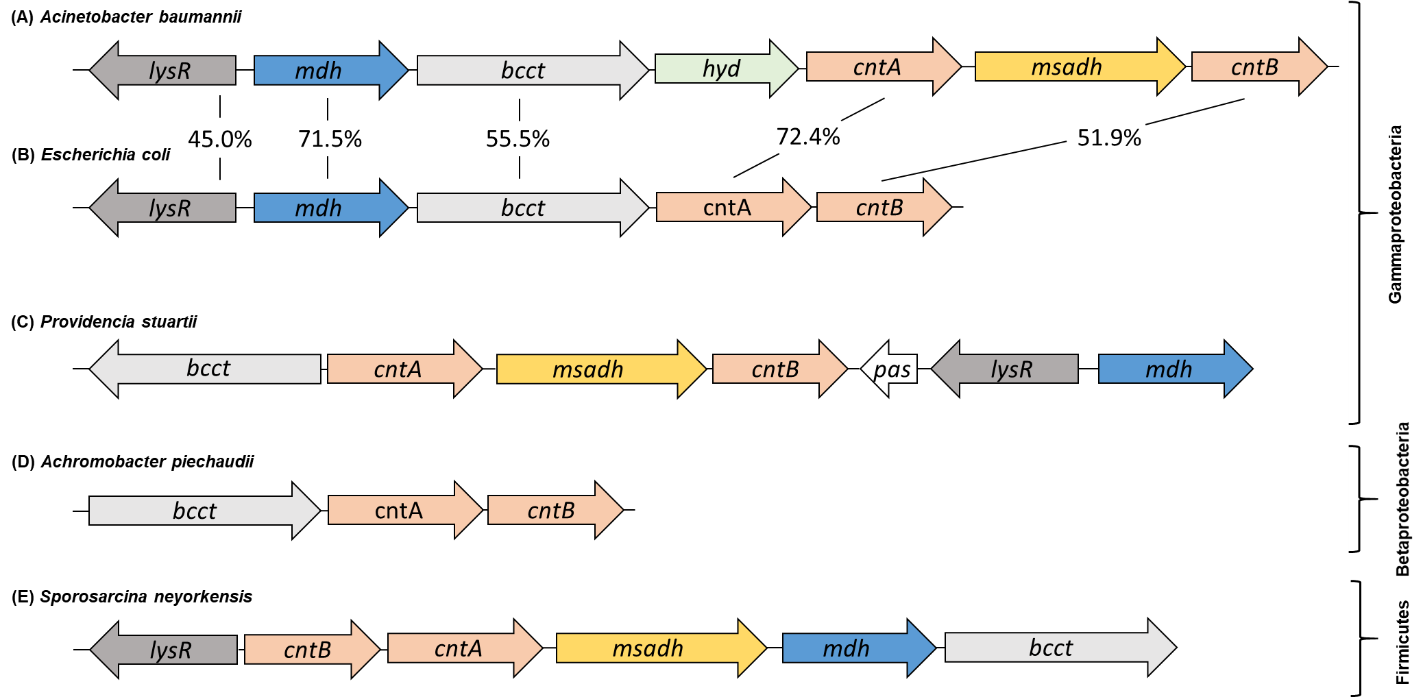


**Figure S5.** **Comparison of the L-carnitine degradation gene operon of *A. baumannii* with related gene clusters in representative genomes of human microbiota.** Sequence identity values for the orthologous genes in *A. baumanni* and *E. coli* are indicated. *lysR*, LysR-type transcriptional regulator also termed *carR*; *mdh*, malate dehydrogenase; *bcct*, betaine/choline/carnitine transporter; *hyd*, postulated hydrolase; *cntA* and *cntB*, subunits of carnitine monooxygenase; *msadh*, malic semialdehyde dehydrogenase and *pas*, postulated PAS domain protein. The figure is adopted from (2).


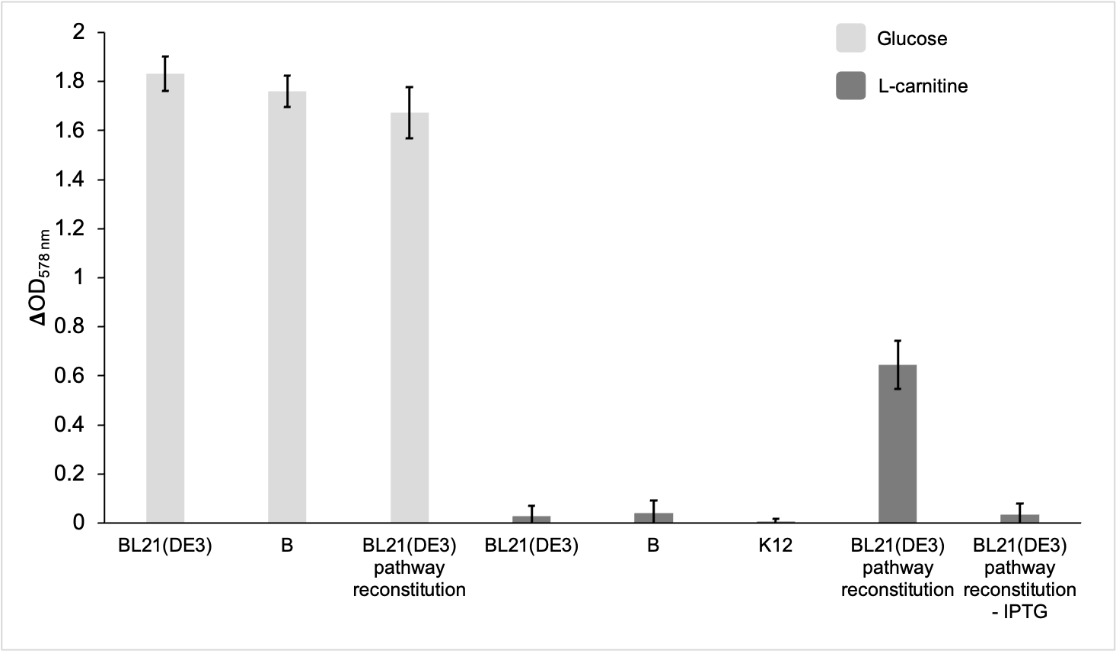


**Figure S6.** **Reconstitution of the carnitine catabolic pathway in *E. coli*.** Growth experiments of *E. coli* strains (BL21(DE3), B or K12) on liquid minimal media containing 20 mM glucose (light *grey bars*) or L-carnitine (*grey bars*) as the sole carbon and energy source were conducted. The increase of the OD_578_ after 24 h incubation is depicted. A pathway reconstitution experiment using strain *E. coli* BL21(DE3) carrying plasmids pETM11-*cntAB*-*bcct* and pACYCDuet-1-*msadh*-*mdh* on L-carnitine was performed. Successful implementation of the pathway in *E. coli* BL21(DE3) was demonstrated by an increase of the OD_578_ of 0.6 after 24 h incubation (BL21(DE3) pathway reconstitution). Control experiments in the presence of glucose or in absence of the inducer IPTG were performed. All experiments were performed in triplicates.

**Table S1: X-ray data collection and refinement statistics.**

| **Structure** | MSA-DH |  |
| --- | --- | --- |
| PDB-ID: | 8S33 |  |
| **Data collection** |  |  |
| Beamline | DESY, Petra III, P11 |  |
| Wavelength (Å) | 1.033 |  |
| Space group | P2_1_2_1_2_1_ |  |
| Cell dimensions |  |  |
| *a*, *b*, *c* (Å) | 115.97, 194.29, 454.34 |  |
| 𝛼, 𝛽, 𝛾 (°) | 90, 90, 90 |  |
| Resolution (Å)^a^ | 19.799 – 2.589 | (2.633 – 2.589) |
| *R*_merge_ (%)^a^ | 15.0 | (145.5) |
| *R*_pim_ (%)^a^ | 4.4 | (42.9) |
| *I*/𝜎*I^a^* | 10.7 | (2.1) |
| Completeness (%)^a^ | 99.7 | (100) |
| Redundancy^a^ | 12.5 | (12.3) |
| CC_1/2_ (%)^a^ | 99.8 | (84.8) |
|  |  |  |
| **Refinement** |  |  |
| Resolution (Å) | 2.59 |  |
| No. reflections | 316280 | (31389) |
| *R*_work_/*R*_free_ (%) | 18.0/20.7 | (26.9/30.0) |
| No. atoms (non-H) | 50929 |  |
| Protein | 49935 |  |
| Ligand/ion | 135 |  |
| Water | 859 |  |
| B-factors (Å^2^) | 60.60 |  |
| Protein | 60.72 |  |
| Ligand/ion | 67.14 |  |
| Water | 52.19 |  |
| R.m.s deviations |  |  |
| Bond lengths (Å) | 0.002 |  |
| Bond angles (º) | 0.459 |  |
| Ramachandran statistics (%) |  |  |
| Favored | 96.22 |  |
| Allowed | 3.71 |  |
| Outliers | 0.07 |  |
| Clashscore (MolProbity) | 0.77 |  |
| MolProbity score | 1.00 |  |

**2 Supplementary Literature**

1 Jurrus, E., et al. (2018) Improvements to the APBS biomolecular solvation software suite. Protein Sci. 27, 112-128 10.1002/pro.3280

2 Zhu, Y., et al. (2014) Carnitine metabolism to trimethylamine by an unusual Rieske-type oxygenase from human microbiota. *Proc. Natl. Acad. Sci. USA*. **111**, 4268-4273 10.1073/pnas.1316569111
